# Radial Extracorporeal Shock Wave Therapy Versus Multimodal Physical Therapy in Non-Traumatic (Degenerative) Rotator Cuff Tendinopathy with Partial Supraspinatus Tear: A Randomized Controlled Trial

**DOI:** 10.3390/jcm15020471

**Published:** 2026-01-07

**Authors:** Zheng Wang, Lan Tang, Ni Wang, Lihua Huang, Christoph Schmitz, Jun Zhou, Yingjie Zhao, Kang Chen, Yanhong Ma

**Affiliations:** 1 Department of Rehabilitation Medicine, Shanghai Sixth People’s Hospital Affiliated to Shanghai Jiao Tong University School of Medicine, Shanghai 200233, China; wangzheng@shsmu.edu.cn (Z.W.); tanglan1995@126.com (L.T.); huanglihua906@163.com (L.H.); 18930172590@163.com (J.Z.); zyj9603240013@163.com (Y.Z.); njmuck@163.com (K.C.); 2 Shanghai YangZhi Rehabilitation Hospital (Shanghai Sunshine Rehabilitation Center), Tongji University School of Medicine, Shanghai 201619, China; wangniwn2021@163.com; 3 Extracorporeal Shock Wave Research Unit, Chair of Neuroanatomy, Institute of Anatomy, Faculty of Medicine, LMU Munich, 80336 Munich, Germany; christoph_schmitz@med.uni-muenchen.de

**Keywords:** shoulder pain, supraspinatus tendon, rotator cuff tendinopathy, partial supraspinatus tear, radial extracorporeal shock wave therapy, rESWT, physical therapy modalities, randomized controlled trial, rehabilitation

## Abstract

**Background/Objectives:** Non-traumatic (degenerative) rotator cuff tendinopathy with partial supraspinatus tear (NT-RCTT) is a common source of shoulder pain and disability. Comparative evidence between radial extracorporeal shock wave therapy (rESWT) and multimodal physical therapy modalities (PTMs) remains scarce. **Methods:** In this single-center randomized controlled trial, 60 adults with MRI-confirmed NT-RCTT were assigned (1:1) to rESWT (one session weekly for six weeks; 2000 impulses per session, 2 bar air pressure, positive energy flux density 0.08 mJ/mm^2^; 8 impulses per second) or a multimodal PTM program (interferential current, shortwave diathermy and magnetothermal therapy; five sessions weekly for six weeks). All participants performed standardized home exercises. The primary outcome was the American Shoulder and Elbow Surgeons (ASES) total score; secondary outcomes included pain (visual analog scale, VAS), satisfaction, range of motion (ROM), supraspinatus tendon (ST) thickness and acromiohumeral distance (AHD). Assessments were conducted at baseline, and at week 6 (W6) and week 12 (W12) post-baseline. **Results:** Both interventions significantly improved all outcomes, but rESWT produced greater and faster effects. Mean ASES total scores increased by 31 ± 5 points with rESWT versus 26 ± 6 with PTMs (*p* < 0.05). VAS pain decreased from 5.2 ± 0.7 to 1.0 ± 0.7 with rESWT and from 5.2 ± 0.8 to 1.7 ± 0.8 with PTMs (*p* < 0.01). rESWT achieved higher satisfaction and larger gains in abduction, flexion and external rotation. Ultrasound showed reduced ST thickness and increased AHD after rESWT but not after PTMs. No serious adverse events occurred. **Conclusions:** rESWT yielded superior pain relief, functional recovery and tendon remodeling compared with a multimodal PTM program, with markedly lower treatment time and excellent tolerability.

## 1. Introduction

Non-traumatic (degenerative) rotator cuff tendinopathy is a leading cause of shoulder pain and functional limitation in adults, with incidence rates reported between 13% and 37% [1]. Among these cases, supraspinatus tendinopathy—with or without partial-thickness tearing—accounts for over 90% of presentations [2,3]. Partial-thickness tears of the supraspinatus tendon (ST), commonly detected by magnetic resonance imaging (MRI) or ultrasonography, are considered part of the degenerative tendinopathy spectrum [2]. Non-traumatic (degenerative) rotator cuff tendinopathy with partial supraspinatus tear (hereafter, NT-RCTT) not only diminishes quality of life but also imposes a significant socioeconomic and healthcare burden [3].

Management of NT-RCTT includes both conservative and surgical approaches. According to the 2019 Clinical Practice Guidelines of the American Academy of Orthopaedic Surgeons (AAOS), surgical repair is primarily recommended for symptomatic full-thickness or acute tears, or when conservative management fails [4]. Most partial-thickness tears and non-calcific tendinopathies are best managed conservatively through exercise therapy and various physical therapy modalities (PTMs), such as interferential current therapy, shortwave diathermy, magnetotherapy, ultrasound, laser therapy and pulsed electromagnetic field therapy (e.g., [5,6,7,8,9,10,11,12,13]), as well as extracorporeal shock wave therapy (ESWT) (reviewed in [14,15,16]). Although many PTMs have shown therapeutic benefits [5,6,7,8,9,10,11,12,13,14,15,16], direct comparative randomized controlled trials (RCTs) evaluating their relative efficacy remain scarce [12,13].

Over the past decades, ESWT has gained prominence as a non-invasive treatment for musculoskeletal disorders [14,15,16]. Two main variants exist: focused ESWT (fESWT) and radial ESWT (rESWT) [14,17]. Both modalities generate acoustic impulses that trigger biological responses leading to pain reduction and tissue remodeling, and appear to operate via similar mechanisms [17]. Their main distinction lies in the pattern of energy distribution and the depth of tissue penetration [14]. Ultrasonographic measurements have shown that the ST is located at an average depth of approximately 9–10 mm beneath the skin surface in individuals with normal body mass index (BMI; i.e., up to 25 kg/m^2^), and around 19 mm in overweight or obese individuals [18]. Even in obese patients, the ST remains within 2 cm of the skin surface [18]. Furthermore, in the hand-in-back position, depth to the ST was reduced by 1.1–8.5 mm [18]. This superficial position places the ST well within the effective penetration range of rESWT [19], making it a suitable and accessible treatment target across different body types. Indeed, evidence supports the safety and efficacy of rESWT in both calcific and non-calcific shoulder tendinopathies [20,21,22]. However, rigorous head-to-head comparisons between rESWT and multimodal PTM programs in NT-RCTT are lacking.

Traditional multimodal PTM programs—combinations of electrotherapeutic and thermal modalities such as interferential current, shortwave diathermy and magnetothermal therapy—are widely employed in rehabilitation settings to reduce inflammation, relieve pain and improve circulation (e.g., [23,24,25,26,27]). Yet, the magnitude and durability of their clinical benefits relative to rESWT remain uncertain. Furthermore, the potential of rESWT to induce structural tendon changes, as reflected in ultrasonographic measures of ST thickness and acromiohumeral distance (AHD), warrants further investigation.

Given the paucity of comparative data, this study aimed to evaluate whether rESWT yields superior clinical and structural outcomes compared with a representative multimodal PTM program in adults with NT-RCTT. We hypothesized that (i) both rESWT and multimodal PTMs would result in significant improvements in shoulder function, pain reduction, patient satisfaction, shoulder range of motion (ROM) and sonographic tendon morphology, but (ii) rESWT would produce superior outcomes without an increased risk of adverse events.

## 2. Materials and Methods

This prospective, single-center randomized controlled trial (RCT) compared rESWT with a multimodal PTM program in adults with NT-RCTT. The trial complied with the Declaration of Helsinki (2024 revision [28]) and the CONSORT 2010 guidelines for RCTs [29]. The study protocol was approved by the Ethics Committee of Shanghai Sixth People’s Hospital affiliated with Shanghai Jiao Tong University School of Medicine (approval No. 2023-132-(1)) and prospectively registered in the Chinese Clinical Trial Register (ChiCTR2300077386; registration date: 7 November 2023). All participants provided written informed consent prior to enrollment.

Eligible participants were adults aged 18–65 years with unilateral shoulder pain lasting at least four weeks. Inclusion criteria were: (1) unilateral shoulder pain with or without restricted shoulder ROM; (2) MRI demonstrating abnormal ST morphology with partial-thickness tear; (3) ultrasonographic confirmation of NT-RCTT on the affected side and a normal contralateral shoulder; and (4) absence of contraindications to rESWT or PTMs. Exclusion criteria included: (1) traumatic or bilateral shoulder symptoms; (2) calcific tendinitis or full-thickness ST tear; (3) combined shoulder pathology (fracture, dislocation or nerve injury); (4) prior local injection therapy; (5) active infection, inflammatory, neoplastic or systemic joint disease (e.g., rheumatoid arthritis); (6) severe osteoporosis or coagulation disorder; (7) presence of a pacemaker or defibrillator, pregnancy or sensory dysfunction; (8) cognitive impairment or psychiatric illness; and (9) refusal to consent or noncompliance with study procedures.

**Figure 1:**
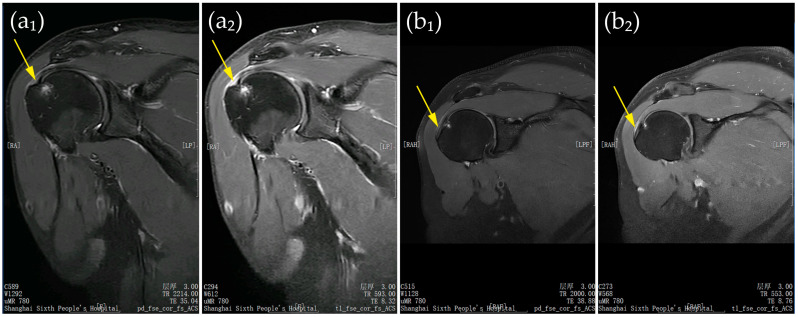
(**a**,**b**) Representative shoulder MRI scans of two patients at baseline. Panels (**a_1_**,**b_1_**) show proton density–weighted fast spin-echo images in the coronal plane with fat saturation and autocalibration for parallel imaging; panels (**a_2_**,**b_2_**) show T1-weighted fast spin-echo images in the same plane with identical parameters. Yellow arrows indicate partial-thickness ST tears. All patient-identifying information has been removed to protect privacy.

Figure 1 presents representative shoulder MRI scans of two patients at baseline.

All diagnoses were made by board-certified rehabilitation physicians with at least ten years of clinical and musculoskeletal imaging experience. MRI and ultrasonography results were reviewed by a blinded musculoskeletal radiologist. The sample size was calculated using G*Power 3.1 [30,31] (test family: F tests; MANOVA, repeated measures, within–between interaction) with α = 0.05 (two-tailed), effect size f = 0.25 (medium), and power = 0.9, yielding 31 participants per group. Allowing for a 20% dropout, a total of 62 participants (31 per group) was required.

A total of 69 participants were randomized, of whom 60 (30 per group) initiated treatment and were included in the modified intention-to-treat (mITT) analysis. Randomization sequences were generated using the SAS PROC PLAN procedure (SAS Studio version 2023.10, SAS Institute, Cary, NC, USA) with a block size of 6. Participants were allocated to two groups: 33 to Group A and 36 to Group B. Allocation codes were sealed in opaque, sequentially numbered envelopes held by an independent coordinator. After baseline assessments, the principal investigator opened the envelope corresponding to each participant’s identification number to confirm group allocation. Three participants in the rESWT group and six in the PTM group withdrew before treatment initiation and were excluded from the mITT analysis. Baseline characteristics of withdrawn participants were compared with those who completed treatment to assess potential bias. Outcome assessors and the musculoskeletal radiologist were blinded to treatment allocation. Because participant and therapist blinding was not feasible, this limitation and its possible influence on subjective outcomes were acknowledged.

Participants assigned to rESWT received six weekly sessions using a Swiss DolorClast rESWT device (Electro Medical Systems, Nyon, Switzerland) with an EvoBlue handpiece and a 15 mm applicator. Each session delivered 2000 radial extracorporeal shock waves (rESWs) at a positive energy flux density (EFD^+^) of 0.08 mJ/mm^2^ (2 bar air pressure; [19]), at a frequency of 8 rESWs per second, for approximately five minutes. Participants were seated with the affected arm placed behind the body (palm to iliac spine), and the treatment site was identified under ultrasound guidance. rESWs were applied circumferentially around the lesion without local anesthesia or sedation.

It should be noted that protocols for rESWT that would represent a consensus in the literature for the treatment of NT-RCTT do not exist. The PEDro database [32]—whose relevance for ESWT research has been established previously [14]—currently lists only seven RCTs investigating ESWT for non-calcific rotator cuff tendinopathy [33,34,35,36,37,38,39], which most closely corresponds to the indication examined in the present study. Only one of these trials used rESWT, whereas the remaining six applied fESWT. Table 1 summarizes the key characteristics of the ESWT treatment protocols used in these studies; Table A1 in Appendix A provides additional details.

One study [37] demonstrated statistically significantly superior outcomes of ESWT compared with sham treatment, no treatment or an alternative therapy (Category 1 in Table 1). Three studies [33,38,39] showed statistically significant clinical improvement from baseline with both ESWT and an alternative treatment, without significant between-group differences at follow-up (Category 2a in Table 1). Two studies [34,35] reported statistically significant improvement with both ESWT and sham or no treatment, again without significant between-group differences at follow-up (Category 2b in Table 1), and one study [36] compared different ESWT protocols (Category 3 in Table 1). No optimal treatment protocol for NT-RCTT could be derived. In light of the negative findings reported in [34,35], the present study employed a commonly used number of impulses per session (2000) and a standard inter-session interval of 7 days, with the EFD set at a level tolerated by all patients in the rESWT group. In contrast to earlier RCTs on non-calcific rotator cuff tendinopathy, which typically used two to four treatment sessions (see Table 1), the number of rESWT sessions was increased to six.

Participants in the PTM group underwent a multimodal PTM program consisting of 30 sessions over six weeks (five sessions per week). Each session included interferential current therapy for 15 min (SK-10WDX, Minato Medical Science Co., Ltd., Osaka, Japan), shortwave diathermy for 10 min (DL-C-M, Shantou Medical Equipment Factory Co., Ltd., Shantou, China) and magnetothermal therapy for 20 min (Hot Magner HM-202, Chuo Medical System Co., Ltd., Tokyo, Japan; low-heat mode).

Protocols for applying a multimodal PTM program consisting of interferential current therapy, shortwave diathermy and magneto-thermal therapy for the treatment of NT-RCTT have not been published. The selection of these modalities and the specific settings used in the present study are based on our own clinical experience and may differ from those used at other institutions.

All participants received health education and home exercise instruction based on the AAOS Rotator Cuff and Shoulder Conditioning Program [40]. In addition, participants who, at any clinic visit, presented with restricted passive ROM or poor tolerance to active or passive shoulder movements underwent passive joint motion (PJM) using a continuous passive motion device (JKJ-1; Canwell Medical Co., Ltd., Jinhua, China). This intervention was implemented to prevent joint adhesions and to maintain or progressively improve shoulder ROM during the early phase of rehabilitation. All participants received at least one PJM session during the six-week treatment period. The number of PJM sessions, as well as the frequency and duration of application, were individualized according to clinical need and patient tolerance. PJM was administered once or twice daily, with each session lasting 20–30 min, for up to five sessions per week. The initial ROM settings were individualized based on the patient’s tolerance and clinical presentation, and the motion arc was progressively increased within a pain-free or minimally painful range; specific starting angles and progression rates therefore varied between patients. PJM was discontinued once the patient’s passive ROM reached functional levels, when the patient was able to effectively participate in active rehabilitation exercises, or when joint stiffness had substantially improved. PJM was not intended to directly facilitate rotator cuff tendon healing. Rather, PJM was expected to primarily influence ROM outcomes, whereas improvements in pain intensity and functional scores were considered to be largely attributable to rESWT or the multimodal PTM program.

Participants were instructed to avoid other rehabilitation treatments during the study. Attendance was recorded at each session, and home exercise compliance was monitored weekly through patient logs and therapist verification; adherence ≥80% was considered satisfactory. No participant received additional analgesic or rehabilitation interventions.

The primary outcome was the American Shoulder and Elbow Surgeons (ASES) Shoulder Score [41,42], which equally weights pain and function (0–100 scale; higher scores indicate better condition). The minimal clinically important difference (MCID) after conservative treatment of rotator cuff injury is 12–17 points [43]. Secondary outcomes included pain intensity on a visual analog scale (VAS, 0–10), patient satisfaction on a 10-point scale (0 = completely dissatisfied, 10 = completely satisfied), active and passive shoulder ROM (abduction, flexion, external rotation and internal rotation) measured using a digital goniometer (JL-360-01, Xinliang Instrument Technology Co., Ltd., Shanghai, China), and ultrasound-based structural parameters including ST thickness and acromiohumeral distance (AHD). Shoulder range of motion was assessed according to the protocol illustrated in Figure 3 of [44]. Specifically, flexion was evaluated in the sagittal plane with the elbow extended (see Figure 3a in [44]); abduction was evaluated in the scapular plane with the elbow extended (see Figure 3b in [44]); and external and internal rotation were evaluated with the shoulder in abduction (see Figure 3d in [44]).

Ultrasound evaluations were performed using a SonoScape E2 system (SonoScape Medical Corp., Shenzhen, China) following standardized protocols. ST thickness was measured at the most affected site and the corresponding contralateral point. AHD was defined as the shortest distance between the inferior acromion and the superior humeral head. Measurements were performed by a blinded musculoskeletal radiologist using a 12 MHz linear probe. All outcomes were assessed at baseline (BL), six weeks post-baseline (W6) and twelve weeks post-baseline (W12).

Statistical analyses were performed using GraphPad Prism (version 10.6.1 for Windows; GraphPad Software, Boston, MA, USA) under the modified intention-to-treat (mITT) framework, including all participants who received at least one treatment session. Data normality was assessed using the D’Agostino–Pearson test. As the majority of datasets for all investigated variables met the normality assumption, mixed-effects models were fitted using restricted maximum likelihood estimation (REML) to compare differences between follow-up time points and between groups. Fixed effects included time (three levels), treatment group (two levels), and the time-by-group interaction, while participants were modeled as random effects to account for repeated measures. For ST thickness, fixed effects also included side (two levels). The sphericity assumption was tested, and when violated, the Geisser–Greenhouse correction was applied. Statistical significance was set at α = 0.05 (two-sided). When significant effects were observed, Bonferroni-adjusted post hoc comparisons were performed for within-group contrasts (baseline vs. W6, baseline vs. W12 and W6 vs. W12) and for between-group differences at each time point to control the familywise error rate. As all participants included in the mITT population completed follow-up assessments, no imputation for missing data was required. The statistical power of the primary endpoint was calculated using OpenEpi [45].

To assess whether the exclusion of patients who refused treatment (and were therefore not included in the mITT population) introduced bias, baseline comparability between treatment groups was evaluated in both the full randomized and the mITT populations. For each baseline variable, standardized mean differences (SMDs) were calculated as the difference in group means divided by the pooled standard deviation for continuous variables, and by the pooled proportion standard error for binary variables [46]. Absolute changes in SMDs between the randomized and mITT populations < 0.1 were interpreted as negligible imbalance and values < 0.2 as acceptable.

## 3. Results

### 3.1. Participant Flow and Baseline Characteristics

Of 69 randomized participants, 60 (30 per group) began treatment and were included in the mITT population. Nine participants (3 in the rESWT group, 6 in the PTMs group) withdrew before starting treatment and were excluded from efficacy analyses. No participant was lost to follow-up after treatment initiation.

**Figure 2:**
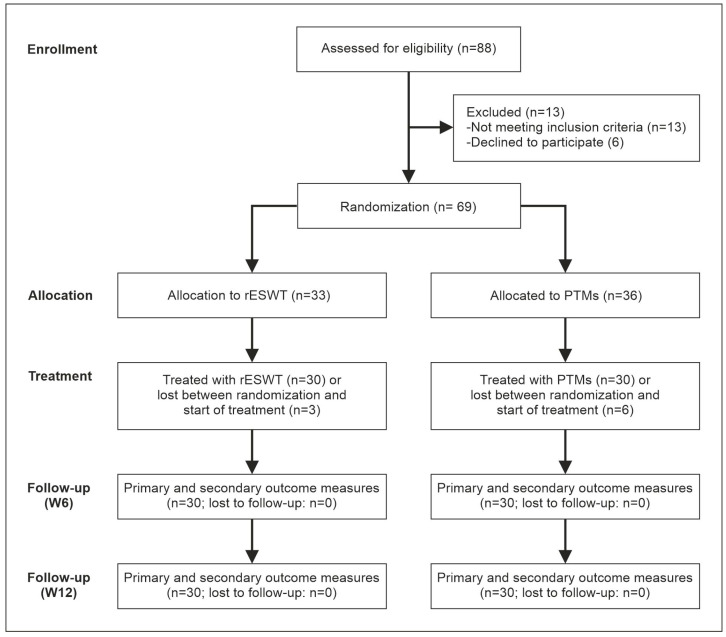
Flow of patients through this trial according to the CONSORT statement [29].

Figure 2 presents the CONSORT flow diagram illustrating enrollment, randomization, allocation, follow-up and analysis.

**Figure 3:**
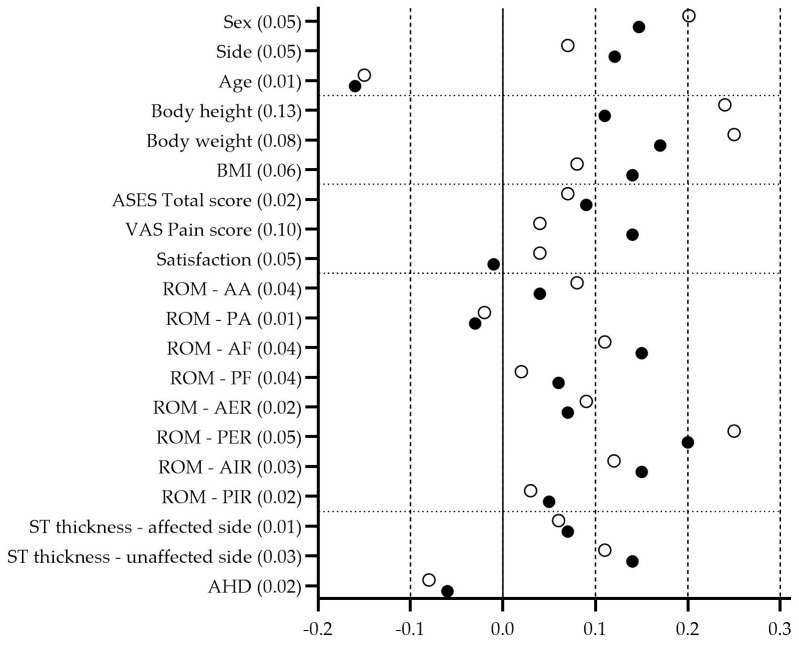
Distribution of baseline SMDs illustrating differences in baseline balance before and after exclusion of patients who refused treatment (black dots: full randomized population, *n* = 69; open dots: mITT population, *n* = 60). Absolute changes in SMDs between the randomized and mITT populations are shown alongside the variable names.

Figure 3 presents a “Love plot” [46] illustrating the distribution of baseline SMDs before and after exclusion of patients who refused treatment.

Baseline characteristics were well balanced between treatment groups in both the full randomized (*n* = 69) and mITT (*n* = 60) populations. SMDs for all baseline variables ranged from −0.20 to +0.25, remaining well below the conventional threshold of 0.3, which indicates good overall balance [46].

Excluding the nine patients who refused treatment resulted in only minor changes in SMDs across all variables (range 0.01–0.13). The largest shift (0.13) occurred for body height, while all other variables showed changes ≤ 0.10. These findings confirm that patient exclusion did not materially affect baseline comparability between treatment groups or introduce detectable selection bias.

The mITT population (*n* = 60) comprised 31 men (51.7%) and 29 women. Right shoulder involvement occurred in 65% of participants. At baseline, the two groups were comparable in demographic and clinical characteristics (Table 2; baseline data of the investigated variables are provided below).

### 3.2. Primary Outcome: ASES Total Score

Table 3 summarizes the ASES Total Score data at baseline and key outcomes at W6 and W12, while Table A2 and Table A3 in Appendix B present the corresponding statistical analysis results.

At baseline, ASES total scores were comparable between groups. Both rESWT and PTM interventions led to progressive improvements at W6 and W12. However, gains were consistently greater in the rESWT group.

By W6, the mean ASES total score had increased from approximately 51 to 68 in the rESWT group, compared with an increase from 51 to 64 in the PTM group. At W12, scores further improved to 82 in the rESWT group and 76 in the PTM group. These changes represent average improvements of about 31 points with rESWT and 26 points with PTMs, both exceeding the MCID for nonoperative treatment of rotator cuff disease [43].

Mixed-effects modeling confirmed significant time-related improvement across both groups and a clear advantage for rESWT, with a steeper recovery trajectory and higher final ASES total scores. Within each group, scores improved steadily from baseline to W6 and continued to rise through W12, indicating sustained functional recovery.

Overall, both treatments enhanced the ASES total score, but rESWT yielded faster and more pronounced improvement, achieving clinically meaningful benefits that were maintained throughout follow-up. The study achieved statistical power of 65.7% at W6 and 92.8% at W12 for detecting this difference (two-sided 95% CI). Consequently, the results led to the rejection of both null hypotheses.

### 3.3. Secondary Outcomes—Pain, Patient Satisfaction with Shoulder Pain and Function, and Shoulder Range of Motion

#### 3.3.1. Pain and Patient Satisfaction with Shoulder Pain and Function

Table 4 summarizes the VAS Pain Score and Patient Satisfaction with Shoulder Pain and Function data at baseline and key outcomes at W6 and W12, while Table A4, Table A5, Table A6 and Table A7 in Appendix B present the corresponding statistical analysis results.

At baseline, pain intensity and satisfaction levels were similar between the two groups. Both treatments led to clear pain reduction and higher satisfaction over time, but the improvements were consistently greater in the rESWT group.

In the rESWT group, mean VAS pain scores decreased from 5.2 at baseline to 3.0 at W6 and 1.0 at W12, while the PTM group showed smaller reductions to 3.5 and 1.7, respectively. These changes represent average improvements of about 4.2 points with rESWT and 3.5 points with PTMs, both exceeding the MCID of 1.4 for nonoperative treatment of rotator cuff disease [43].

Patient satisfaction with shoulder pain and function rose from about 4.4 at baseline to 6.7 at W6 and 8.4 at W12 with rESWT, compared with increases to 6.0 and 7.6 after PTMs. To date, no MCID has been established for patient satisfaction with shoulder pain and function in the context of nonoperative management of rotator cuff disease.

Mixed-effects modeling indicated that both interventions produced substantial time-dependent improvement, with rESWT demonstrating a steeper and more pronounced trajectory for both pain relief and satisfaction. Within-group comparisons showed steady progress from baseline through W12, confirming sustained benefit in each group.

Overall, both therapies effectively reduced pain and improved patient satisfaction with shoulder pain and function, but rESWT provided faster and greater relief and higher overall satisfaction by the end of follow-up.

#### 3.3.2. Shoulder Range of Motion

Table 5 summarizes the Shoulder ROM data at baseline and key outcomes at W6 and W12, while Table A8, Table A9, Table A10, Table A11, Table A12, Table A13, Table A14, Table A15, Table A16, Table A17, Table A18, Table A19, Table A20, Table A21, Table A22 and Table A23 in Appendix B present the corresponding statistical analysis results.

At baseline, all active and passive shoulder ROM measures were comparable between groups. Both treatments produced significant improvements in shoulder mobility over time, but rESWT consistently resulted in greater and faster gains across most movements.

By W6, the rESWT group showed larger increases in active and passive abduction and flexion, with further progression through W12. Mean active abduction rose from about 99 degrees to 132 degrees at W6 and 158 degrees at W12 with rESWT, compared with 98 degrees to 120 degrees and 143 degrees in the PTM group. Similar patterns were observed for flexion and external rotation, where rESWT maintained a clear advantage. Internal rotation improved in both groups, though differences were smaller. An MCID for shoulder ROM measures following nonoperative treatment of rotator cuff disease has not been established in the literature.

Mixed-effects modeling confirmed a marked effect of time in both interventions and an overall superior recovery pattern with rESWT. Improvements progressed continuously from baseline to W12, indicating sustained mobility restoration.

In summary, both treatments enhanced shoulder ROM, but rESWT achieved greater and more rapid recovery, particularly in abduction, flexion and external rotation, aligning with its superior functional and pain outcomes.

### 3.4. Structural Ultrasound Outcomes—Supraspinatus Tendon Thickness and Acromiohumeral Distance

#### 3.4.1. Supraspinatus Tendon Thickness

**Figure 4:**
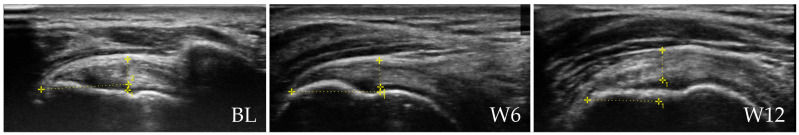
Representative ultrasound images of the supraspinatus tendon (ST) of a patient in the rESWT group at baseline (BL), 6 weeks (W6) and 12 weeks (W12) post-baseline. ST thickness measurements are indicated by vertical lines (5.7 mm at BL, 5.1 mm at W6 and 5.1 mm at W12). All patient-identifying information has been removed to protect privacy.

Figure 4 presents representative ultrasound images of the ST of a patient from the rESWT group at BL, W6 and W12.

Table 6 summarizes all ST Thickness data at baseline and key outcomes at W6 and W12, while Table A24 and Table A25 in Appendix B present the corresponding statistical analysis results.

At baseline, ST thickness was comparable between groups on both the affected and contralateral shoulders. Over the 12-week follow-up, both treatments produced measurable changes, but the pattern differed markedly between groups.

In the rESWT group, the affected-side ST showed a gradual decrease in mean thickness from approximately 5.6 mm at baseline to 5.5 mm at W6 and 5.4 mm at W12, while the contralateral, unaffected side remained stable. In contrast, the PTM group exhibited minimal or no change across time on either side. To date, no MCID has been established for ST thickness in the context of nonoperative management of rotator cuff disease.

Mixed-effects modeling confirmed that only participants receiving rESWT experienced a meaningful reduction in ST thickness, whereas ST thickness remained essentially unchanged in the PTM group.

Between-group comparisons further supported this finding, showing that the difference in ST thickness reduction between the affected and contralateral sides was greater after rESWT than after PTMs at both follow-up points. The continuous narrowing of the tendon in the rESWT group suggests a trend toward normalization of tendon morphology and possible resolution of degenerative swelling.

Taken together, these results indicate that rESWT not only improved clinical symptoms but was also associated with measurable structural remodeling of the ST, an effect not observed with the multimodal PTM program.

#### 3.4.2. Acromiohumeral Distance

**Figure 5:**
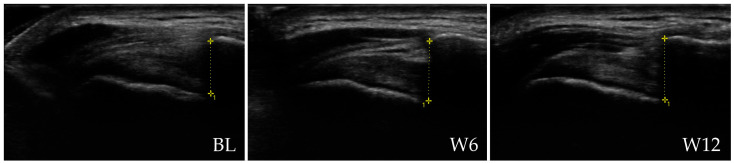
Representative ultrasound images of the shoulder of a patient from the rESWT group at baseline (BL), 6 weeks (W6) and 12 weeks (W12) post-baseline, illustrating AHD measurements indicated by vertical lines (10.3 mm at BL, 10.9 mm at W6 and 11.0 mm at W12). All patient-identifying information has been removed to protect privacy.

Figure 5 presents representative ultrasound images of the shoulder of a patient from the rESWT group at BL, W6 and W12, illustrating changes in AHD.

Table 7 summarizes the Acromiohumeral Distance data at baseline and key outcomes at W6 and W12, while Table A26 and Table A27 in Appendix B present the corresponding statistical analysis results.

At baseline, the AHD was similar between groups, indicating comparable initial shoulder structure. Over the 12-week period, both groups showed minor changes, but only the rESWT group demonstrated a consistent and measurable increase.

In the rESWT group, mean AHD rose from 10.8 mm at baseline to 11.0 mm at W6 and 11.1 mm at W12, whereas values in the PTM group remained essentially unchanged throughout follow-up. An MCID for AHD measures following nonoperative treatment of rotator cuff disease has not been established in the literature.

Mixed-effects modeling confirmed a clear time-related increase in AHD after rESWT, with no corresponding trend in the PTM group.

These findings suggest that rESWT may promote subtle structural improvements within the subacromial space, potentially reflecting reduced tendon impingement and improved shoulder mechanics.

### 3.5. Safety and Adverse Events

No serious or unexpected adverse events were reported. Minor transient local discomfort (mild erythema or soreness) occurred in three rESWT participants and resolved spontaneously within 24 h. No participant discontinued treatment due to adverse effects.

## 4. Discussion

In this RCT, rESWT provided superior pain relief, functional improvement, structural restoration and time efficiency compared with a multimodal PTM program in adults with NT-RCTT. Both interventions produced significant clinical benefits, but rESWT consistently yielded greater and faster improvements in ASES total scores, pain reduction, patient satisfaction and shoulder ROM, along with measurable sonographic changes—namely, reduced ST thickness and increased AHD. These results reinforce rESWT as an effective and well-tolerated conservative therapy for partial rotator cuff injury.

The mean ASES improvement in the rESWT group exceeded the MCID [35] by more than twofold at W12, confirming both statistical and clinical significance. Pain intensity decreased by approximately 80% from baseline, and satisfaction ratings rose by nearly 90% during the same period, indicating that patients experienced rapid and meaningful relief. Additionally, shoulder ROM improved across all planes of motion, with especially pronounced gains in abduction and flexion. These functional enhancements were supported by sonographic evidence of structural recovery—ST thickness reduction and subacromial spacing increase—suggesting that rESWT not only alleviates symptoms but may also promote biological tendon remodeling. Importantly, these benefits were achieved through only six short treatment sessions, totaling roughly 30 min of contact time, compared with approximately 1350 min for the multimodal PTM program, highlighting the remarkable time efficiency of rESWT for both patients and clinicians.

The present findings align with and extend the existing literature on the therapeutic value of rESWT for shoulder tendinopathies. Several studies have confirmed the safety and efficacy of rESWT in managing calcific and non-calcific shoulder disorders, including subacromial impingement and rotator cuff tendinopathy [20,21,22]. However, these trials left the relative efficacy of rESWT versus multimodal PTMs unclear. By directly comparing rESWT with a multimodal PTM program comprising interferential current therapy, shortwave diathermy and magnetothermal therapy—three commonly applied modalities in clinical rehabilitation [6,7,8,12,23,24,25,26,27]—this study provides robust comparative evidence that rESWT delivers superior short-term outcomes in pain, function and tendon morphology.

The magnitude and consistency of functional improvement observed after rESWT are particularly notable. Mean ASES total scores increased by approximately 31 points in the rESWT group compared with 26 points in the PTM group, with both groups surpassing the MCID [43] but with significantly larger gains in the rESWT group. These improvements were accompanied by greater reductions in VAS pain and higher patient satisfaction. The improvement trajectory suggests that the effects of rESWT continue to progress beyond the active treatment phase, as indicated by further gains between W6 and W12. This sustained recovery pattern supports the hypothesis that rESWT initiates biological and neuromuscular processes conducive to long-term tendon healing rather than merely producing transient analgesia [17,47,48].

The ultrasonographic findings of this study provide valuable insight into the mechanisms underlying the observed clinical outcomes. rESWT was associated with a significant reduction in ST thickness and a measurable increase in AHD, whereas the PTM group exhibited no notable structural changes. The reduction in tendon thickness following rESWT likely reflects decreased intratendinous edema and improved collagen alignment, consistent with tissue normalization processes demonstrated in experimental and histological studies. Specifically, animal and in vitro research has shown that both rESWT and fESWT facilitate the resolution of inflammatory infiltration and edema, enhance tenocyte proliferation and type I collagen synthesis, and restore a well-organized collagen network [47,48,49,50,51]. These structural adaptations correspond with imaging findings from human studies, which have reported reduced tendon thickness and improved echotexture after rESWT [52,53] (Notably, in [53], a combined rESWT/fESWT protocol was described; however, the device used—BTL 5000 SWT Power; BTL, Prague, Czech Republic—generates only rESWs). Collectively, these observations suggest that post-treatment reductions in tendon size reflect normalization of tendon morphology and extracellular matrix organization (c.f. [54,55,56]).

Furthermore, the observed increase in AHD may indicate reduced mechanical impingement of the ST beneath the acromion, as greater AHD is associated with lower subacromial pressure [57], whereas reduced AHD correlates with ST pathology and subacromial impingement [58,59]. Accordingly, the greater AHD observed in this study may relate to the improvements in shoulder abduction and flexion range of motion. Together, these findings suggest that rESWT not only alleviates pain and enhances shoulder mobility but also promotes tendon remodeling and subacromial decompression, thereby contributing to improved joint biomechanics.

Several mechanisms may explain the therapeutic effects of rESWT on tendinopathies (summarized in [17]). In vivo studies using the same rESWT device as in the present study showed a rapid, transient upregulation of pro-regenerative inflammatory mediators, particularly IL-6 and IL-8, together with increased expression of pro-forms of MMP-2 and MMP-9 in healthy and tendinopathic human Achilles tendons [47]. These factors regulate extracellular matrix turnover and angiogenesis, suggesting that rESWT induces a controlled catabolic–anabolic response that supports tendon remodeling [47]. In addition, rESWT-induced mechanical stress activates pathways involved in connective tissue repair, including NF-κB modulation, fibroblast proliferation, improved collagen organization and increased vascularization in animal and cell models (reviewed in [17]). These effects align with the structural and clinical improvements observed in the present study. Furthermore, fESWT has been shown to reduce substance P levels, thereby attenuating pain and neurogenic inflammation [60], and there is no evidence that this effect would not also occur after rESWT.

Although direct histological evidence in human ST tissue remains limited, the parallel clinical and ultrasonographic improvements demonstrated here are well aligned with these mechanistic insights.

From a clinical perspective, rESWT offers several practical advantages. It is non-invasive, well-tolerated and highly time-efficient. Its short treatment duration can enhance patient compliance and reduce healthcare resource utilization, making it an attractive alternative or adjunct to conventional physiotherapy in busy rehabilitation settings. Moreover, the absence of serious adverse events and the rapid resolution of mild local reactions confirm its excellent safety profile.

The present findings are consistent with earlier reports indicating that rESWT can enhance the effects of exercise-based rehabilitation. In a recent RCT on patients with unilateral shoulder impingement syndrome [61], exercise-based rehabilitation (EBR) consisting of shoulder mobilizations, scapular muscle exercises and rotator cuff strengthening was compared with EBR combined with a single corticosteroid injection and with EBR combined with rESWT (three sessions, one per week) using the same rESWT device as in the present study. The diagnosis of shoulder impingement was made clinically, based on a stage II Neer classification and at least two positive provocative tests (Neer, painful arc, Hawkins–Kennedy, or external rotation), without imaging confirmation. At 12 weeks post-baseline, the rESWT group achieved greater improvements in pain, disability and shoulder ROM than either comparator group, with no major adverse effects reported.

In both [61] and the current trial, the subacromial space was assessed as a structural outcome. However, no significant change in this parameter was detected in [61], which may be related to the shorter treatment course of only three rESWT sessions. In contrast, the present study—conducted in patients with NT-RCTT—demonstrated a measurable increase in AHD alongside marked clinical improvement. Despite differences in diagnostic criteria and inclusion parameters, both studies clearly showed that adding rESWT results in superior outcomes compared with exercise-based therapy alone. The present findings extend those of [61] by providing evidence of concurrent structural tendon recovery, supporting the reproducibility of rESWT’s therapeutic effects across related shoulder disorders.

Further evidence for the synergistic potential of rESWT with exercise comes from a study on chronic midportion Achilles tendinopathy, in which eccentric loading combined with rESWT—again using the same device—produced greater pain and function improvements than eccentric loading alone after four months [62]. Although participants in both groups of the present trial performed standardized home exercises, the superior outcomes achieved with rESWT suggest complementary biological and functional effects between shock wave–induced tissue remodeling and exercise-driven adaptation. Future studies should explore optimized combined protocols—such as rESWT paired with targeted strengthening or proprioceptive training—to maximize long-term recovery and minimize recurrence.

This study possesses several notable strengths. It employed a prospective randomized controlled design with blinded outcome assessment and strict adherence to CONSORT guidelines [29]. Both interventions were protocolized and delivered by experienced therapists, ensuring consistency and reproducibility. The inclusion of ultrasound-based structural measures adds objective evidence of morphological change, an element often lacking in prior ESWT studies. Treatment adherence was excellent, and no participant was lost to follow-up, minimizing attrition bias.

Nevertheless, certain methodological limitations must be acknowledged. First, treatment frequency and total exposure time differed markedly between groups. Although this reflects real-world practice—where multimodal PTM programs are typically more time-intensive—it introduces a potential performance bias related to treatment duration and therapist contact. Second, participant and therapist blinding was not feasible due to the nature of the interventions, which may have influenced subjective outcomes such as pain and satisfaction. Third, although the mITT approach strengthened internal validity by including only participants who initiated treatment, it excluded a small number of randomized subjects, introducing a minimal risk of post-randomization bias.

Additionally, while Bonferroni correction was applied to control for multiple comparisons, the analysis of numerous secondary endpoints increased the risk of type I error. The relatively short 12-week follow-up period limits the assessment of long-term durability, and MRI confirmation of tendon changes was not performed, restricting structural validation to ultrasound findings. Moreover, the absence of a sham or placebo control group means that nonspecific effects related to rESWT cannot be entirely excluded.

Despite these limitations, the present trial provides compelling short-term evidence that rESWT is more effective and efficient than a conventional multimodal PTM program for NT-RCTT. Future multicenter RCTs should include sham or time-matched comparator arms to further reduce bias and strengthen causal inference. Longer follow-up periods are necessary to determine the persistence of both clinical and structural benefits. Integration of MRI-based morphologic assessments would enhance the validity of imaging outcomes, and the addition of biochemical or elastographic markers could elucidate underlying repair mechanisms. Future studies should also explore the role of rESWT as part of a multimodal rehabilitation strategy combining physical, biological and exercise-based interventions.

## 5. Conclusions

This study demonstrates that rESWT may be associated with faster, greater and more comprehensive recovery than a multimodal PTM program in patients with NT-RCTT. The observed improvements in pain, function, ROM and tendon morphology, together with minimal treatment time and good tolerability, indicate that rESWT could represent an efficient and patient-friendly conservative treatment option for partial rotator cuff injury. While these findings support the potential clinical value of rESWT, they should be interpreted as preliminary evidence that warrants further investigation, particularly with respect to long-term outcomes and the underlying biological mechanisms.

## Figures and Tables

**Table 1:** Key characteristics of the ESWT treatment protocols used in RCTs on non-calcific rotator cuff tendinopathy listed in the PEDro database as of 14 December 2025; additional details are provided in Table A1 in Appendix A.

R	Y	F/R	S	I	N	EFD	T_EFD_	F	OC
[33]	2001	F	3	7	2000	0.33 mJ/mm^2^	A	NR	2a
[34]	2001	F	3	7	2000	0.11 mJ/mm^2^	A	NR	2b
[35]	2002	F	3	28	1500	0.12 mJ/mm^2^	B	NR	2b
[36]	2009	F	3	7	2000	0.78 vs. 0.44 mJ/mm^2^	A	NR	3
[37]	2012	F	2	7	3000	0.068 mJ/mm^2^	B	NR	1
[38]	2017	F	4	7	1600	0.15 mJ/mm^2^	B	4	2a
[39]	2023	R	3	7	2000	4 bar	C	5	2a

Abbreviations: RCT, randomized controlled trial; R, reference; Y, year of publication; F/R, use of fESWT (F) or rESWT (R); S, number of treatment sessions; I, interval between treatment sessions [days]; N, number of impulses per treatment session; EFD; energy flux density; T_EFD_, type of energy flux density (categories of T_EFD_: A, positive EFD; B, not specified whether the reported EFD was the positive EFD or the total EFD; C, air pressure, but not the EFD of the rESWs reported); F, frequency at which the impulses were applied [Hertz] (NR, not reported); O, outcome category (categories of O: Category 1: superior outcomes of ESWT compared with sham treatment, no treatment or an alternative therapy; Category 2a: statistically significant clinical improvement from baseline with both ESWT and an alternative treatment, without significant between-group differences at follow-up; Category 2b: statistically significant improvement with both ESWT and sham or no treatment, again without significant between-group differences at follow-up; Category 3: comparison between different ESWT protocols.

**Table 2:** Baseline demographic and clinical characteristics of the patients (mITT population) enrolled in this trial (baseline data of the investigated variables are provided below).

	Min	25%	Median	75%	Max	Mean	SD
Age [years]							
rESWT	20	32	42.5	49	58	40.2	10.5
PTMs	22	33.5	40.5	51.3	61	41.8	11.1
Body height [cm]							
rESWT	150	161	168.5	175	190	168.0	9.1
PTMs	150	158	164.5	175	183	165.9	9.2
Body weight [kg]							
rESWT	50	55.8	61	70	80	62.5	8.1
PTMs	49	54.5	60	66.3	79	60.5	8.1
BMI [kg/m^2^]							
rESWT	18.8	20.8	22.2	23.2	25.9	22.1	1.7
PTMs	17.0	21.0	21.9	23.2	26.3	21.9	1.9

Abbreviations: Min, minimum; 25%, 25% percentile; 75%, 75% percentile; SD, standard deviation; rESWT, radial extracorporeal shock wave therapy; PTMs, physical therapy modalities; BMI, body mass index.

**Table 3:** ASES Total Score data at baseline and key outcomes at W6 and W12.

	rESWT			PTMs			95% CI of Diff.	*p* Value
	Median	Mean	SD	Median	Mean	SD		
BL	50.5	51.0	5.3	49.0	50.6	5.6	−2.4 to 3.2	
W6	68.0	67.7	6.6	61.5	63.7	6.4	0.6 to 7.3	0.021
W12	83.0	81.5	5.4	76.0	76.1	6.9	2.3 to 8. 7	0.001

Abbreviations: rESWT, radial extracorporeal shock wave therapy; PTMs, physical therapy modalities; 95% CI of diff., 95% confidence interval of mean difference; SD, standard deviation; BL, baseline; W6, week 6 post-baseline; W12, week 12 post-baseline. All datasets met the normality assumption.

**Table 4:** VAS Pain Score and Patient Satisfaction with Shoulder Pain and Function data at baseline and key outcomes at W6 and W12.

	rESWT			PTMs			95% CI of Diff.	*p* Value
	Median	Mean	SD	Median	Mean	SD		
VAS Pain Score								
BL	5.0	5.2	0.7	5.0	5.2	0.8	−0.4 to 0.4	
W6	3.0	3.0	0.7	3.5	3.5	0.7	−0.9 to −0.1	0.011
W12	1.0	1.0	0.7	2.0	1.7	0.8	−1.1 to −0.3	0.001
Patient Satisfaction with Shoulder Pain and Function								
BL	4.5	4.4	0.8	4.5	4.4	0.9	−0.4 to 0.5	
W6	7.0	6. 7	0.7	6.0	6.0	0.8	0.3 to 1.0	0.002
W12	8.2	8.4	0.6	7.8	7.6	0.7	0.5 to 1.2	<0.001

Abbreviations: rESWT, radial extracorporeal shock wave therapy; PTMs, physical therapy modalities; 95% CI of diff., 95% confidence interval of mean difference; SD, standard deviation; BL, baseline; W6, week 6 post-baseline; W12, week 12 post-baseline. All datasets met the normality assumption.

**Table 5:** Shoulder ROM data [degrees] at baseline and key outcomes at W6 and W12.

	rESWT			PTMs			95% CI of Diff.	*p* Value
	Median	Mean	SD	Median	Mean	SD		
Active Abduction								
BL	96.7 *	99.1 *	17.4 *	93.6	97.7	18.3	−7.8 to 10.7	
W6	133.5	132.3	19.1	120.8	119.9	18.4	2.7 to 22.1	0.013
W12	160.3 *	157.9 *	13.9 *	141.8	143.2	16.6	6.8 to 22.6	<0.001
Passive Abduction								
BL	114.3 *	113.7 *	15.6 *	112.3	113.9	16.8	−8.6 to 8.1	
W6	145.7	146.0	15.3	133.3	134.7	16.9	2.9 to 19.5	0.009
W12	172.8 *	170.8 *	8.5 *	154.3	156.2	13.9	8.6 to 20.5	<0.001
Active Flexion								
BL	109.6	117.3	24.5	110.0	114.5	24.9	−10.0 to 15.6	
W6	156.1 *	154.1 *	17.0 *	146.0	144.3	18.8	0.6 to 19.1	0.037
W12	173.4	172.8	5.7	168.5 *	164.7 *	12.7 *	2.9 to 13.2	0.003
Passive Flexion								
BL	126.3	133.3	23.4	130.5	132.8	23.2	−11.6 to 12.5	
W6	167.7 *	165.6 *	12.9 *	156.5	157.0	16.0	1.2 to 16.2	0.024
W12	178.6	177.3	2.9	172.9 *	170.6 *	9.0 *	3.2 to 10.2	<0.001
Active External Rotation								
BL	43.3	44.1	8.2	41.5	43.3	8.8	−3.7 to 5.2	
W6	67.0	66.0	9.4	59.3	60.0	9.8	1.1 to 11.0	0.018
W12	79.2 *	78.9 *	7.8 *	77.1 *	74.2 *	8.7 *	0.5 to 9.0	0.031
Passive External Rotation								
BL	53.9	54.2	8.7	52.3	52.2	7.8	−2.2 to 6.3	
W6	74.8	74.0	7.6	67.8	67.9	8.1	2.1 to 10.3	0.003
W12	86.0 *	84.5 *	5.7 *	83.1 *	79.7 *	8.3 *	1.1 to 8.5	0.012
Active Internal Rotation								
BL	35.4	34.9	7.4	32.9	34.0	8.4	−3.2 to 5.0	
W6	50.8	51.0	6.1	49.6	49.8	6.6	−2.1 to 4.5	0.473
W12	59.8	60.4	4.1	57.8 *	57.1 *	5.3 *	0.9 to 5.7	0.009
Passive Internal Rotation								
BL	44.6	44.1	8.4	42.8	43.9	7.8	−4.0 to 4.4	
W6	59.7 *	58.3 *	8.2 *	56.8	57.0	6.1	−2.4 to 5.1	0.477
W12	68.2 *	67.4 *	3.3 *	63.1	62.9	4.7	2.4 to 6.6	<0.001

Abbreviations: rESWT, radial extracorporeal shock wave therapy; PTMs, physical therapy modalities; 95% CI of diff., 95% confidence interval of mean difference; SD, standard deviation; BL, baseline; W6, week 6 post-baseline; W12, week 12 post-baseline. Datasets that did not meet the normality assumption are indicated by asterisks; all remaining datasets met this assumption.

**Table 6:** Supraspinatus Tendon Thickness data [mm] at baseline and key outcomes at W6 and W12.

		rESWT			PTMs			95% CI of Diff.	*p* Value
		Median	Mean	SD	Median	Mean	SD		
BL	a	5.8 *	5.6 *	0.8 *	5.7	5.7	0.8	−0.7 to 0.8	
	c	5.3	5.2	0.6	5.0	5.1	0.7	−0.5 to 0.7	
95% CI of diff.			0.0 to 1.2			0.0 to 1.1			
*p* Value			0.007			0.023			
W6	a	5.5	5.5	0.5	5.7	5.7	0.8	−0.8 to 0.4	0.975
	c	5.3	5.2	0.6	5.0	5.1	0.7	−0.5 to 0.6	>0.999
95% CI of diff.			0.0 to 0.6			0.1 to 1.1			
*p* Value			0.135			0.016			
W12	a	5.4	5.4	0.5	5.7	5.7	0.8	−0.9 to 0.3	0.895
	c	5.2	5.2	0.6	5.1	5.1	0.7	−0.5 to 0.6	>0.999
95% CI of diff.			−0.1 to 0.5			0.0 to 1.1			
*p* Value			0.815			0.027			

Abbreviations: rESWT, radial extracorporeal shock wave therapy; PTMs, physical therapy modalities; 95% CI of diff., 95% confidence interval of mean difference; SD, standard deviation; BL, baseline; W6, week 6 post-baseline; W12, week 12 post-baseline; a, affected side; c, contralateral (unaffected) side. Baseline data from the affected side did not meet the normality assumption (indicated by asterisks), whereas all other datasets met this assumption.

**Table 7:** Acromiohumeral Distance data [mm] at baseline, as well as corresponding key outcomes at W6 and W12.

	rESWT			PTMs			95% CI of Diff.	*p* Value
	Median	Mean	SD	Median	Mean	SD		
BL	10.8	10.8	0.9	10.9	10.8	0.9	−0.5 to 0.4	
W6	11.0	11.0	0.8	10.9	10.8	0.9	−0.3 to 0.6	0.472
W12	11.0	11.1	0.8	10.7 *	10.8 *	0.9 *	−0.2 to 0.7	0.251

Abbreviations: rESWT, radial extracorporeal shock wave therapy; PTMs, physical therapy modalities; 95% CI of diff., 95% confidence interval of mean difference; SD, standard deviation; BL, baseline; W6, week 6 post-baseline; W12, week 12 post-baseline; a, affected side; c, contralateral (unaffected) side. Data from the PTMs group at W12 did not meet the normality assumption (indicated by asterisks), whereas all other datasets met this assumption.

## Data Availability

The anonymized datasets generated and analyzed during this study are available from the corresponding author upon reasonable request and in compliance with institutional and data protection regulations.

## Abbreviations

The following abbreviations are used in this manuscript:

NT-RCTT	Non-traumatic rotator cuff tendinopathy/tear
rESWT	Radial extracorporeal shock wave therapy
PTMs	Physical therapy modalities
ASES	American Shoulder and Elbow Surgeons
VAS	Visual analogue scale
RCT	Randomized controlled trial
ROM	Range of motion
ST	Supraspinatus tendon
AHD	Acromiohumeral distance
MRI	Magnetic resonance imaging
AAOS	American Academy of Orthopaedic Surgeons
ESWT	Extracorporeal shock wave therapy
fESWT	Focused extracorporeal shock wave therapy
BMI	Body mass index
ChiCTR	Chinese Clinical Trial Register
mITT	modified Intention-To-Treat
rESWs	Radial extracorporeal shock waves
EFD^+^	Positive energy flux density
PJM	Passive joint motion
MCID	Minimal clinically important difference
BL	Baseline
W6	Week 6 post-baseline
W12	Week 12 post-baseline
REML	Restricted maximum likelihood (estimation)
SMDs	Standardized mean differences
CONSORT	Consolidated Standards of Reporting Trials
Min	Minimum
25%	25% percentile
75%	75% percentile
SD	Standard deviation
95% CI of diff.	95% confidence interval of mean difference
a	Affected side
c	Contralateral (unaffected) side
DF	Degrees of freedom
SE	Standard error
α	Significance level
IL	Interleukin
MMP	Matrix metalloproteinase
NF-κB	Nuclear factor kappa-B
EBR	Exercise-based rehabilitation

## Appendix A

**Table A1:** Additional details on studies of non-calcific rotator cuff tendinopathy listed in the PEDro database [32] as of 14 December 2025.

R	Device	Manufacturer	One-Sentence Summary of the Study
[33]	Minilith SL1	Storz Medical (Tägerwillen, Switzerland)	fESWT was compared with x-ray stimulation therapy and showed no clinically relevant benefit for non-calcific supraspinatus tendinopathy.
[34]	Minilith SL1	Storz Medical	fESWT showed no advantage over sham treatment in patients with non-calcific supraspinatus tendinopathy.
[35]	Sonocur Plus	Siemens (Erlangen, Germany)	fESWT for non-calcific tendinopathy of the rotator cuff produced no greater benefit than sham treatment, with improvements in both groups likely due to a significant placebo effect.
[36]	Minilith SL1	Storz Medical	fESWT was compared with low-energy fESWT, showing similar clinical outcomes in non-calcific rotator cuff tendinopathy.
[37]	Modulith SLK	Storz Medical	fESWT demonstrated superior short-term clinical benefits compared with placebo for treating non-calcific rotator cuff tendinopathy.
[38]	Modulith SLK	Storz Medical	fESWT produced significant short-term improvements in pain and shoulder function that were comparable to those achieved with hyaluronic acid injections in patients with non-calcific rotator cuff tendinopathy.
[39]	Masterpuls 100	Storz Medical	rESWT was compared with corticosteroid injection for non-calcific rotator cuff tendinopathy, demonstrating similar short-term clinical effectiveness.

Abbreviations: fESWT, focused extracorporeal shock wave therapy; rESWT, radial extracorporeal shock wave therapy.

## Appendix B

Abbreviations in all tables in Appendix B: CI, confidence interval; DF, degree of freedom; diff., difference; SD, standard deviation; SE, standard error.

**Table A2:** Summary of mixed-effects model results for ASES Total Score outcomes.

Mixed-effects model (REML)	Matching:	Stacked		
	Assume sphericity?	No		
	Alpha	0.05		
Fixed effects (type III)	*p* value	F (DFn, DFd)	Geisser-Greenhouse’s epsilon	
Time	<0.001	F (1.809, 104.9) = 915.9	0.9045	
Treatment	0.02	F (1, 58) = 5.751		
Time × Treatment	<0.001	F (1.809, 104.9) = 7.884	0.9045	
Random effects	SD	Variance		
Subject	4.871	23.72		
Residual	3.59	12.89		
Was the matching effective?	Chi-square, DF	72.91, 1	*p* value	<0.001
Difference between column means	Predicted mean of rESWT		66.72	
	Predicted mean of PTMs		63.44	
	Difference between predicted means		3.278	
	SE of difference		1.367	
	95% CI of difference		0.5419 to 6.014	
Data summary	Number of columns (Treatment)		2	
	Number of rows (Time)		3	
	Number of subjects (Subject)		60	
	Number of missing values		0	
Compare cell means with others in its row and its column				
Number of families		5		
Number of comparisons per row family		1		
Number of comparisons per column family		3		

**Table A3:** Summary of the results from Bonferroni’s multiple comparison tests for ASES Total Score outcomes.

Bonferroni’s multiple comparisons test		Mean diff.	95% CI of diff.	Adjusted *p* Value			
BL	rESWT vs. PTMs	0.4	−2.399 to 3.199	0.776			
W6	rESWT vs. PTMs	3.967	0.607 to 7.327	0.021			
W12	rESWT vs. PTMs	5.467	2.265 to 8.668	0.001			
rESWT	BL vs. W6	−16.7	−19.12 to −14.28	<0.001			
	BL vs. W12	−30.57	−33.15 to −27.98	<0.001			
	W6 vs. W12	−13.87	−15.93 to −11.80	<0.001			
PTMs	BL vs. W6	−13.13	−15.56 to −10.71	<0.001			
	BL vs. W12	−25.5	−28.18 to −22.82	<0.001			
	W6 vs. W12	−12.37	−14.22 to −10.51	<0.001			
Test details		Mean 1	Mean 2	Mean diff.	SE of diff.	t	DF
BL	rESWT vs. PTMs	50.97	50.57	0.4	1.398	0.2861	57.81
W6	rESWT vs. PTMs	67.67	63.7	3.967	1.678	2.363	57.93
W12	rESWT vs. PTMs	81.53	76.07	5.467	1.597	3.423	54.66
rESWT	BL vs. W6	50.97	67.67	−16.7	0.952	17.54	29
	BL vs. W12	50.97	81.53	−30.57	1.018	30.03	29
	W6 vs. W12	67.67	81.53	−13.87	0.813	17.05	29
PTMs	BL vs. W6	50.57	63.7	−13.13	0.954	13.77	29
	BL vs. W12	50.57	76.07	−25.5	1.053	24.21	29
	W6 vs. W12	63.7	76.07	−12.37	0.730	16.95	29

**Table A4:** Summary of mixed-effects model results for VAS Pain Score outcomes.

Mixed-effects model (REML)	Matching:	Stacked		
	Assume sphericity?	No		
	Alpha	0.05		
Fixed effects (type III)	*p* value	F (DFn, DFd)	Geisser-Greenhouse’s epsilon	
Time	<0.001	F (1.777, 103.1) = 746.0	0.8887	
Treatment	0.019	F (1, 58) = 5.788		
Time × Treatment	0.003	F (1.777, 103.1) = 6.728	0.8887	
Random effects	SD	Variance		
Subject	0.5201	0.2705		
Residual	0.546	0.2981		
Was the matching effective?	Chi-square, DF	36.13, 1	*p* value	<0.001
Difference between column means	Predicted mean of rESWT		3.089	
	Predicted mean of PTMs		3.467	
	Difference between predicted means		−0.3778	
	SE of difference		0.157	
	95% CI of difference		−0.6921 to −0.06346	
Data summary	Number of columns (Treatment)		2	
	Number of rows (Time)		3	
	Number of subjects (Subject)		60	
	Number of missing values		0	
Compare cell means with others in its row and its column				
Number of families		5		
Number of comparisons per row family		1		
Number of comparisons per column family		3		

**Table A5:** Summary of the results from Bonferroni’s multiple comparison tests for VAS Pain Score outcomes.

Bonferroni’s multiple comparisons test		Mean diff.	95% CI of diff.	Adjusted *p* Value			
BL	rESWT vs. PTMs	0.03333	−0.363 to 0.430	0.867			
W6	rESWT vs. PTMs	−0.5	−0.881 to −0.119	0.011			
W12	rESWT vs. PTMs	−0.6667	−1.058 to −0.275	0.001			
rESWT	BL vs. W6	2.233	1.918 to 2.548	<0.001			
	BL vs. W12	4.2	3.807 to 4.593	<0.001			
	W6 vs. W12	1.967	1.681 to 2.252	<0.001			
PTMs	BL vs. W6	1.7	1.398 to 2.002	<0.001			
	BL vs. W12	3.5	3.065 to 3.935	<0.001			
	W6 vs. W12	1.8	1.407 to 2.193	<0.001			
Test details		Mean 1	Mean 2	Mean diff.	SE of diff.	t	DF
BL	rESWT vs. PTMs	5.233	5.2	0.033	0.198	0.1682	57.42
W6	rESWT vs. PTMs	3	3.5	−0.5	0.190	2.628	57.99
W12	rESWT vs. PTMs	1.033	1.7	−0.667	0.196	3.409	57.42
rESWT	BL vs. W6	5.233	3	2.233	0.124	18.02	29
	BL vs. W12	5.233	1.033	4.2	0.155	27.16	29
	W6 vs. W12	3	1.033	1.967	0.112	17.52	29
PTMs	BL vs. W6	5.2	3.5	1.7	0.119	14.3	29
	BL vs. W12	5.2	1.7	3.5	0.171	20.44	29
	W6 vs. W12	3.5	1.7	1.8	0.155	11.64	29

**Table A6:** Summary of mixed-effects model results for Patient Satisfaction with Shoulder Pain and Function outcomes.

Mixed-effects model (REML)	Matching:	Stacked		
	Assume sphericity?	No		
	Alpha	0.05		
Fixed effects (type III)	*p* value	F (DFn, DFd)	Geisser-Greenhouse’s epsilon	
Time	<0.001	F (1.759, 102.0) = 578.5	0.8795	
Treatment	0.002	F (1, 58) = 10.58		
Time × Treatment	0.001	F (1.759, 102.0) = 7.567	0.8795	
Random effects	SD	Variance		
Subject	0.4839	0.2341		
Residual	0.5732	0.3285		
Was the matching effective?	Chi-square, DF	27.29, 1	*p* value	<0.001
Difference between column means	Predicted mean of rESWT		6.492	
	Predicted mean of PTMs		6	
	Difference between predicted means		0.4922	
	SE of difference		0.1514	
	95% CI of difference		0.1893 to 0.7952	
Data summary	Number of columns (Treatment)		2	
	Number of rows (Time)		3	
	Number of subjects (Subject)		60	
	Number of missing values		0	
Compare cell means with others in its row and its column				
Number of families		5		
Number of comparisons per row family		1		
Number of comparisons per column family		3		

**Table A7:** Summary of the results from Bonferroni’s multiple comparison tests for Patient Satisfaction with Shoulder Pain and Function outcomes.

Bonferroni’s multiple comparisons test		Mean diff.	95% CI of diff.	Adjusted *p* Value			
BL	rESWT vs. PTMs	0.03333	−0.399 to 0.466	0.878			
W6	rESWT vs. PTMs	0.6333	0.252 to 1.015	0.002			
W12	rESWT vs. PTMs	0.81	0.466 to 1.154	<0.001			
rESWT	BL vs. W6	−2.233	−2.667 to −1.799	<0.001			
	BL vs. W12	−3.943	−4.382 to −3.505	<0.001			
	W6 vs. W12	−1.71	−2.038 to −1.382	<0.001			
PTMs	BL vs. W6	−1.633	−1.967 to −1.300	<0.001			
	BL vs. W12	−3.167	−3.581 to −2.752	<0.001			
	W6 vs. W12	−1.533	−1.812 to −1.255	<0.001			
Test details		Mean 1	Mean 2	Mean diff.	SE of diff.	t	DF
BL	rESWT vs. PTMs	4.433	4.4	0.0333	0.2159	0.1544	57.88
W6	rESWT vs. PTMs	6.667	6.033	0.633	0.1907	3.321	57.69
W12	rESWT vs. PTMs	8.377	7.567	0.81	0.1719	4.713	55.86
rESWT	BL vs. W6	4.433	6.667	−2.233	0.1708	13.08	29
	BL vs. W12	4.433	8.377	−3.943	0.1725	22.86	29
	W6 vs. W12	6.667	8.377	−1.71	0.129	13.26	29
PTMs	BL vs. W6	4.4	6.033	−1.633	0.1312	12.45	29
	BL vs. W12	4.4	7.567	−3.167	0.1632	19.41	29
	W6 vs. W12	6.033	7.567	−1.533	0.1097	13.98	29

**Table A8:** Summary of mixed-effects model results for ROM—Active Abduction outcomes.

Mixed-effects model (REML)	Matching:	Stacked		
	Assume sphericity?	No		
	Alpha	0.05		
Fixed effects (type III)	*p* value	F (DFn, DFd)	Geisser-Greenhouse’s epsilon	
Time	<0.001	F (1.617, 93.76) = 815.3	0.8083	
Treatment	0.028	F (1, 58) = 5.069		
Time × Treatment	<0.001	F (1.617, 93.76) = 15.01	0.8083	
Random effects	SD	Variance		
Subject	15.85	251.3		
Residual	7.08	50.12		
Was the matching effective?	Chi-square, DF	151.2, 1	*p* value	<0.001
Difference between column means	Predicted mean of rESWT		129.8	
	Predicted mean of PTMs		120.3	
	Difference between predicted means		9.516	
	SE of difference		4.227	
	95% CI of difference		1.055 to 17.98	
Data summary	Number of columns (Treatment)		2	
	Number of rows (Time)		3	
	Number of subjects (Subject)		60	
	Number of missing values		0	
Compare cell means with others in its row and its column				
Number of families		5		
Number of comparisons per row family		1		
Number of comparisons per column family		3		

**Table A9:** Summary of the results from Bonferroni’s multiple comparison tests for ROM—Active Abduction outcomes.

Bonferroni’s multiple comparisons test		Mean diff.	95% CI of diff.	Adjusted *p* Value			
BL	rESWT vs. PTMs	1.446	−7.772 to 10.66	0.755			
W6	rESWT vs. PTMs	12.4	2.706 to 22.09	0.013			
W12	rESWT vs. PTMs	14.7	6.785 to 22.62	<0.001			
rESWT	BL vs. W6	−33.14	−37.15 to −29.14	<0.001			
	BL vs. W12	−58.79	−64.94 to −52.64	<0.001			
	W6 vs. W12	−25.65	−31.01 to −20.28	<0.001			
PTMs	BL vs. W6	−22.19	−25.11 to −19.27	<0.001			
	BL vs. W12	−45.54	−50.34 to −40.73	<0.001			
	W6 vs. W12	−23.35	−27.24 to −19.45	<0.001			
Test details		Mean 1	Mean 2	Mean diff.	SE of diff.	t	DF
BL	rESWT vs. PTMs	99.13	97.68	1.446	4.605	0.314	57.85
W6	rESWT vs. PTMs	132.3	119.9	12.4	4.843	2.56	57.93
W12	rESWT vs. PTMs	157.9	143.2	14.7	3.952	3.72	56.23
rESWT	BL vs. W6	99.13	132.3	−33.14	1.575	21.04	29
	BL vs. W12	99.13	157.9	−58.79	2.422	24.28	29
	W6 vs. W12	132.3	157.9	−25.65	2.111	12.15	29
PTMs	BL vs. W6	97.68	119.9	−22.19	1.15	19.3	29
	BL vs. W12	97.68	143.2	−45.54	1.89	24.09	29
	W6 vs. W12	119.9	143.2	−23.35	1.534	15.22	29

**Table A10:** Summary of mixed-effects model results for ROM—Passive Abduction outcomes.

Mixed-effects model (REML)	Matching:	Stacked		
	Assume sphericity?	No		
	Alpha	0.05		
Fixed effects (type III)	*p* value	F (DFn, DFd)	Geisser-Greenhouse’s epsilon	
Time	<0.001	F (1.913, 110.9) = 491.1	0.9564	
Treatment	0.014	F (1, 58) = 6.462		
Time × Treatment	<0.001	F (1.913, 110.9) = 11.97	0.9564	
Random effects	SD	Variance		
Subject	11.94	142.5		
Residual	8.69	75.51		
Was the matching effective?	Chi-square, DF	74.50, 1	*p* value	<0.001
Difference between column means	Predicted mean of rESWT		143.5	
	Predicted mean of PTMs		135	
	Difference between predicted means		8.499	
	SE of difference		3.343	
	95% CI of difference		1.807 to 15.19	
Data summary	Number of columns (Treatment)		2	
	Number of rows (Time)		3	
	Number of subjects (Subject)		60	
	Number of missing values		0	
Compare cell means with others in its row and its column				
Number of families		5		
Number of comparisons per row family		1		
Number of comparisons per column family		3		

**Table A11:** Summary of the results from Bonferroni’s multiple comparison tests for ROM—Passive Abduction outcomes.

Bonferroni’s multiple comparisons test		Mean diff.	95% CI of diff.	Adjusted *p* Value			
BL	rESWT vs. PTMs	−0.2533	−8.640 to 8.133	0.952			
W6	rESWT vs. PTMs	11.2	2.886 to 19.52	0.009			
W12	rESWT vs. PTMs	14.55	8.584 to 20.51	<0.001			
rESWT	BL vs. W6	−32.26	−36.79 to −27.73	<0.001			
	BL vs. W12	−57.08	−63.78 to −50.38	<0.001			
	W6 vs. W12	−24.82	−30.35 to −19.29	<0.001			
PTMs	BL vs. W6	−20.8	−26.36 to −15.24	<0.001			
	BL vs. W12	−42.28	−47.72 to −36.84	<0.001			
	W6 vs. W12	−21.48	−27.68 to −15.27	<0.001			
Test details		Mean 1	Mean 2	Mean diff.	SE of diff.	t	DF
BL	rESWT vs. PTMs	113.7	113.9	−0.253	4.189	0.061	57.71
W6	rESWT vs. PTMs	146	134.7	11.2	4.155	2.697	57.46
W12	rESWT vs. PTMs	170.8	156.2	14.55	2.965	4.906	47.91
rESWT	BL vs. W6	113.7	146	−32.26	1.784	18.08	29
	BL vs. W12	113.7	170.8	−57.08	2.637	21.64	29
	W6 vs. W12	146	170.8	−24.82	2.175	11.41	29
PTMs	BL vs. W6	113.9	134.7	−20.8	2.188	9.505	29
	BL vs. W12	113.9	156.2	−42.28	2.14	19.76	29
	W6 vs. W12	134.7	156.2	−21.48	2.442	8.794	29

**Table A12:** Summary of mixed-effects model results for ROM—Active Flexion outcomes.

Mixed-effects model (REML)	Matching:	Stacked		
	Assume sphericity?	No		
	Alpha	0.05		
Fixed effects (type III)	*p* value	F (DFn, DFd)	Geisser-Greenhouse’s epsilon	
Time	<0.001	F (1.637, 94.95) = 293.9	0.8185	
Treatment	0.092	F (1, 58) = 2.928		
Time × Treatment	0.253	F (1.637, 94.95) = 1.388	0.8185	
Random effects	SD	Variance		
Subject	14.02	196.6		
Residual	12.07	145.6		
Was the matching effective?	Chi-square, DF	54.75, 1	*p* value	<0.001
Difference between column means	Predicted mean of rESWT		148.1	
	Predicted mean of PTMs		141.2	
	Difference between predicted means		6.916	
	SE of difference		4.042	
	95% CI of difference		−1.175 to 15.01	
Data summary	Number of columns (Treatment)		2	
	Number of rows (Time)		3	
	Number of subjects (Subject)		60	
	Number of missing values		0	
Compare cell means with others in its row and its column				
Number of families		5		
Number of comparisons per row family		1		
Number of comparisons per column family		3		

**Table A13:** Summary of the results from Bonferroni’s multiple comparison tests for ROM—Active Flexion outcomes.

Bonferroni’s multiple comparisons test		Mean diff.	95% CI of diff.	Adjusted *p* Value			
BL	rESWT vs. PTMs	2.804	−9.952 to 15.56	0.662			
W6	rESWT vs. PTMs	9.859	0.6124 to 19.10	0.037			
W12	rESWT vs. PTMs	8.087	2.938 to 13.24	0.003			
rESWT	BL vs. W6	−36.77	−45.23 to −28.31	<0.001			
	BL vs. W12	−55.46	−65.75 to −45.18	<0.001			
	W6 vs. W12	−18.69	−24.98 to −12.40	<0.001			
PTMs	BL vs. W6	−29.72	−36.79 to −22.65	<0.001			
	BL vs. W12	−50.18	−58.69 to −41.67	<0.001			
	W6 vs. W12	−20.46	−26.52 to −14.41	<0.001			
Test details		Mean 1	Mean 2	Mean diff.	SE of diff.	t	DF
BL	rESWT vs. PTMs	117.3	114.5	2.804	6.372	0.44	57.98
W6	rESWT vs. PTMs	154.1	144.3	9.859	4.618	2.135	57.43
W12	rESWT vs. PTMs	172.8	164.7	8.087	2.548	3.174	40.21
rESWT	BL vs. W6	117.3	154.1	−36.77	3.33	11.04	29
	BL vs. W12	117.3	172.8	−55.46	4.048	13.7	29
	W6 vs. W12	154.1	172.8	−18.69	2.475	7.551	29
PTMs	BL vs. W6	114.5	144.3	−29.72	2.782	10.68	29
	BL vs. W12	114.5	164.7	−50.18	3.349	14.98	29
	W6 vs. W12	144.3	164.7	−20.46	2.383	8.588	29

**Table A14:** Summary of mixed-effects model results for ROM—Passive Flexion outcomes.

Mixed-effects model (REML)	Matching:	Stacked		
	Assume sphericity?	No		
	Alpha	0.05		
Fixed effects (type III)	*p* value	F (DFn, DFd)	Geisser-Greenhouse’s epsilon	
Time	<0.001	F (1.458, 84.58) = 190.9	0.7292	
Treatment	0.127	F (1, 58) = 2.395		
Time × Treatment	0.155	F (1.458, 84.58) = 1.985	0.7292	
Random effects	SD	Variance		
Subject	11.32	128.2		
Residual	11.74	137.7		
Was the matching effective?	Chi-square, DF	37.18, 1	*p* value	<0.001
Difference between column means	Predicted mean of rESWT		158.7	
	Predicted mean of PTMs		153.5	
	Difference between predicted means		5.274	
	SE of difference		3.407	
	95% CI of difference		−1.547 to 12.09	
Data summary	Number of columns (Treatment)		2	
	Number of rows (Time)		3	
	Number of subjects (Subject)		60	
	Number of missing values		0	
Compare cell means with others in its row and its column				
Number of families		5		
Number of comparisons per row family		1		
Number of comparisons per column family		3		

**Table A15:** Summary of the results from Bonferroni’s multiple comparison tests for ROM—Passive Flexion outcomes.

Bonferroni’s multiple comparisons test		Mean diff.	95% CI of diff.	Adjusted *p* Value			
BL	rESWT vs. PTMs	0.4803	−11.56 to 12.52	0.937			
W6	rESWT vs. PTMs	8.669	1.160 to 16.18	0.024			
W12	rESWT vs. PTMs	6.671	3.175 to 10.17	<0.001			
rESWT	BL vs. W6	−32.33	−40.37 to −24.29	<0.001			
	BL vs. W12	−43.98	−54.39 to −33.58	<0.001			
	W6 vs. W12	−11.65	−17.26 to −6.048	<0.001			
PTMs	BL vs. W6	−24.14	−31.26 to −17.02	<0.001			
	BL vs. W12	−37.79	−46.43 to −29.16	<0.001			
	W6 vs. W12	−13.65	−18.78 to −8.526	<0.001			
Test details		Mean 1	Mean 2	Mean diff.	SE of diff.	t	DF
BL	rESWT vs. PTMs	133.3	132.8	0.4803	6.015	0.080	58
W6	rESWT vs. PTMs	165.6	157	8.669	3.748	2.313	55.47
W12	rESWT vs. PTMs	177.3	170.6	6.671	1.722	3.873	35.08
rESWT	BL vs. W6	133.3	165.6	−32.33	3.163	10.22	29
	BL vs. W12	133.3	177.3	−43.98	4.094	10.74	29
	W6 vs. W12	165.6	177.3	−11.65	2.207	5.282	29
PTMs	BL vs. W6	132.8	157	−24.14	2.801	8.617	29
	BL vs. W12	132.8	170.6	−37.79	3.398	11.12	29
	W6 vs. W12	157	170.6	−13.65	2.017	6.767	29

**Table A16:** Summary of mixed-effects model results for ROM—Active External Rotation outcomes.

Mixed-effects model (REML)	Matching:	Stacked		
	Assume sphericity?	No		
	Alpha	0.05		
Fixed effects (type III)	*p* value	F (DFn, DFd)	Geisser-Greenhouse’s epsilon	
Time	<0.001	F (1.786, 103.6) = 804.4	0.8931	
Treatment	0.069	F (1, 58) = 3.424		
Time × Treatment	0.007	F (1.786, 103.6) = 5.570	0.8931	
Random effects	SD	Variance		
Subject	7.57	57.31		
Residual	4.507	20.31		
Was the matching effective?	Chi-square, DF	102.9, 1	*p* value	<0.001
Difference between column means	Predicted mean of rESWT		62.98	
	Predicted mean of PTMs		59.15	
	Difference between predicted means		3.824	
	SE of difference		2.067	
	95% CI of difference		−0.3127 to 7.962	
Data summary	Number of columns (Treatment)		2	
	Number of rows (Time)		3	
	Number of subjects (Subject)		60	
	Number of missing values		0	
Compare cell means with others in its row and its column				
Number of families		5		
Number of comparisons per row family		1		
Number of comparisons per column family		3		

**Table A17:** Summary of the results from Bonferroni’s multiple comparison tests for ROM—Active External Rotation outcomes.

Bonferroni’s multiple comparisons test		Mean diff.	95% CI of diff.	Adjusted *p* Value			
BL	rESWT vs. PTMs	0.742	−3.665 to 5.149	0.737			
W6	rESWT vs. PTMs	6.011	1.055 to 10.97	0.018			
W12	rESWT vs. PTMs	4.72	0.4499 to 8.991	0.031			
rESWT	BL vs. W6	−21.91	−24.35 to −19.46	<0.001			
	BL vs. W12	−34.83	−38.13 to −31.52	<0.001			
	W6 vs. W12	−12.92	−15.75 to −10.09	<0.001			
PTMs	BL vs. W6	−16.64	−19.56 to −13.71	<0.001			
	BL vs. W12	−30.85	−34.40 to −27.30	<0.001			
	W6 vs. W12	−14.21	−16.73 to −11.69	<0.001			
Test details		Mean 1	Mean 2	Mean diff.	SE of diff.	t	DF
BL	rESWT vs. PTMs	44.07	43.33	0.742	2.201	0.3371	57.7
W6	rESWT vs. PTMs	65.97	59.96	6.011	2.476	2.428	57.86
W12	rESWT vs. PTMs	78.89	74.17	4.72	2.133	2.213	57.21
rESWT	BL vs. W6	44.07	65.97	−21.91	0.963	22.74	29
	BL vs. W12	44.07	78.89	−34.83	1.301	26.78	29
	W6 vs. W12	65.97	78.89	−12.92	1.114	11.6	29
PTMs	BL vs. W6	43.33	59.96	−16.64	1.152	14.45	29
	BL vs. W12	43.33	74.17	−30.85	1.398	22.07	29
	W6 vs. W12	59.96	74.17	−14.21	0.992	14.32	29

**Table A18:** Summary of mixed-effects model results for ROM—Passive External Rotation outcomes.

Mixed-effects model (REML)	Matching:	Stacked		
	Assume sphericity?	No		
	Alpha	0.05		
Fixed effects (type III)	*p* value	F (DFn, DFd)	Geisser-Greenhouse’s epsilon	
Time	<0.001	F (1.746, 101.3) = 480.9	0.8729	
Treatment	0.012	F (1, 58) = 6.680		
Time × Treatment	0.092	F (1.746, 101.3) = 2.524	0.8729	
Random effects	SD	Variance		
Subject	5.793	33.56		
Residual	5.152	26.54		
Was the matching effective?	Chi-square, DF	51.31, 1	*p* value	<0.001
Difference between column means	Predicted mean of rESWT		70.93	
	Predicted mean of PTMs		66.58	
	Difference between predicted means		4.346	
	SE of difference		1.681	
	95% CI of difference		0.9800 to 7.711	
Data summary	Number of columns (Treatment)		2	
	Number of rows (Time)		3	
	Number of subjects (Subject)		60	
	Number of missing values		0	
Compare cell means with others in its row and its column				
Number of families		5		
Number of comparisons per row family		1		
Number of comparisons per column family		3		

**Table A19:** Summary of the results from Bonferroni’s multiple comparison tests for ROM—Passive External Rotation outcomes.

Bonferroni’s multiple comparisons test		Mean diff.	95% CI of diff.	Adjusted *p* Value			
BL	rESWT vs. PTMs	2.041	−2.213 to 6.295	0.341			
W6	rESWT vs. PTMs	6.193	2.129 to 10.26	0.003			
W12	rESWT vs. PTMs	4.803	1.110 to 8.495	0.012			
rESWT	BL vs. W6	−19.82	−22.83 to −16.82	<0.001			
	BL vs. W12	−30.3	−33.89 to −26.71	<0.001			
	W6 vs. W12	−10.48	−13.56 to −7.398	<0.001			
PTMs	BL vs. W6	−15.67	−19.29 to −12.05	<0.001			
	BL vs. W12	−27.54	−31.77 to −23.31	<0.001			
	W6 vs. W12	−11.87	−14.36 to −9.383	<0.001			
Test details		Mean 1	Mean 2	Mean diff.	SE of diff.	t	DF
BL	rESWT vs. PTMs	54.22	52.18	2.041	2.125	0.961	57.27
W6	rESWT vs. PTMs	74.04	67.85	6.193	2.03	3.051	57.79
W12	rESWT vs. PTMs	84.52	79.72	4.803	1.84	2.611	51.3
rESWT	BL vs. W6	54.22	74.04	−19.82	1.184	16.75	29
	BL vs. W12	54.22	84.52	−30.3	1.412	21.46	29
	W6 vs. W12	74.04	84.52	−10.48	1.213	8.64	29
PTMs	BL vs. W6	52.18	67.85	−15.67	1.424	11.01	29
	BL vs. W12	52.18	79.72	−27.54	1.663	16.56	29
	W6 vs. W12	67.85	79.72	−11.87	0.979	12.13	29

**Table A20:** Summary of mixed-effects model results for ROM—Active Internal Rotation outcomes.

Mixed-effects model (REML)	Matching:	Stacked		
	Assume sphericity?	No		
	Alpha	0.05		
Fixed effects (type III)	*p* value	F (DFn, DFd)	Geisser-Greenhouse’s epsilon	
Time	<0.001	F (1.477, 85.69) = 521.1	0.7387	
Treatment	0.209	F (1, 58) = 1.613		
Time × Treatment	0.239	F (1.477, 85.69) = 1.451	0.7387	
Random effects	SD	Variance		
Subject	4.943	24.43		
Residual	4.185	17.51		
Was the matching effective?	Chi-square, DF	56.52, 1	*p* value	<0.001
Difference between column means	Predicted mean of rESWT		48.76	
	Predicted mean of PTMs		46.95	
	Difference between predicted means		1.804	
	SE of difference		1.421	
	95% CI of difference		−1.039 to 4.647	
Data summary	Number of columns (Treatment)		2	
	Number of rows (Time)		3	
	Number of subjects (Subject)		60	
	Number of missing values		0	
Compare cell means with others in its row and its column				
Number of families		5		
Number of comparisons per row family		1		
Number of comparisons per column family		3		

**Table A21:** Summary of the results from Bonferroni’s multiple comparison tests for ROM—Active Internal Rotation outcomes.

Bonferroni’s multiple comparisons test		Mean diff.	95% CI of diff.	Adjusted *p* Value			
BL	rESWT vs. PTMs	0.923	−3.179 to 5.025	0.654			
W6	rESWT vs. PTMs	1.19	−2.105 to 4.485	0.473			
W12	rESWT vs. PTMs	3.299	0.8579 to 5.740	0.009			
rESWT	BL vs. W6	−16.09	−19.41 to −12.77	<0.001			
	BL vs. W12	−25.46	−28.87 to −22.05	<0.001			
	W6 vs. W12	−9.367	−10.90 to −7.837	<0.001			
PTMs	BL vs. W6	−15.82	−18.48 to −13.16	<0.001			
	BL vs. W12	−23.08	−26.12 to −20.04	<0.001			
	W6 vs. W12	−7.258	−9.236 to −5.279	<0.001			
Test details		Mean 1	Mean 2	Mean diff.	SE of diff.	t	DF
BL	rESWT vs. PTMs	34.91	33.98	0.923	2.049	0.451	57.12
W6	rESWT vs. PTMs	51	49.81	1.19	1.646	0.723	57.63
W12	rESWT vs. PTMs	60.36	57.06	3.299	1.218	2.709	54.33
rESWT	BL vs. W6	34.91	51	−16.09	1.306	12.32	29
	BL vs. W12	34.91	60.36	−25.46	1.342	18.97	29
	W6 vs. W12	51	60.36	−9.367	0.602	15.56	29
PTMs	BL vs. W6	33.98	49.81	−15.82	1.047	15.11	29
	BL vs. W12	33.98	57.06	−23.08	1.197	19.29	29
	W6 vs. W12	49.81	57.06	−7.258	0.779	9.321	29

**Table A22:** Summary of mixed-effects model results for ROM—Passive Internal Rotation outcomes.

Mixed-effects model (REML)	Matching:	Stacked		
	Assume sphericity?	No		
	Alpha	0.05		
Fixed effects (type III)	*p* value	F (DFn, DFd)	Geisser-Greenhouse’s epsilon	
Time	<0.001	F (1.886, 109.4) = 242.9	0.943	
Treatment	0.129	F (1, 58) = 2.367		
Time × Treatment	0.08	F (1.886, 109.4) = 2.629	0.943	
Random effects	SD	Variance		
Subject	4.074	16.6		
Residual	5.315	28.25		
Was the matching effective?	Chi-square, DF	21.48, 1	*p* value	<0.001
Difference between column means	Predicted mean of rESWT		56.6	
	Predicted mean of PTMs		54.58	
	Difference between predicted means		2.026	
	SE of difference		1.317	
	95% CI of difference		−0.610 to 4.662	
Data summary	Number of columns (Treatment)		2	
	Number of rows (Time)		3	
	Number of subjects (Subject)		60	
	Number of missing values		0	
Compare cell means with others in its row and its column				
Number of families		5		
Number of comparisons per row family		1		
Number of comparisons per column family		3		

**Table A23:** Summary of the results from Bonferroni’s multiple comparison tests for ROM—Passive Internal Rotation outcomes.

Bonferroni’s multiple comparisons test		Mean diff.	95% CI of diff.	Adjusted *p* Value			
BL	rESWT vs. PTMs	0.2327	−3.970 to 4.435	0.912			
W6	rESWT vs. PTMs	1.33	−2.394 to 5.054	0.477			
W12	rESWT vs. PTMs	4.516	2.398 to 6.635	<0.001			
rESWT	BL vs. W6	−14.17	−18.05 to −10.29	<0.001			
	BL vs. W12	−23.23	−27.25 to −19.22	<0.001			
	W6 vs. W12	−9.06	−12.63 to −5.485	<0.001			
PTMs	BL vs. W6	−13.08	−16.51 to −9.642	<0.001			
	BL vs. W12	−18.95	−22.36 to −15.53	<0.001			
	W6 vs. W12	−5.873	−8.235 to −3.511	<0.001			
Test details		Mean 1	Mean 2	Mean diff.	SE of diff.	t	DF
BL	rESWT vs. PTMs	44.13	43.9	0.233	2.099	0.111	57.65
W6	rESWT vs. PTMs	58.31	56.98	1.33	1.857	0.716	53.45
W12	rESWT vs. PTMs	67.37	62.85	4.516	1.056	4.277	52.13
rESWT	BL vs. W6	44.13	58.31	−14.17	1.526	9.285	29
	BL vs. W12	44.13	67.37	−23.23	1.579	14.71	29
	W6 vs. W12	58.31	67.37	−9.06	1.407	6.44	29
PTMs	BL vs. W6	43.9	56.98	−13.08	1.351	9.675	29
	BL vs. W12	43.9	62.85	−18.95	1.344	14.1	29
	W6 vs. W12	56.98	62.85	−5.873	0.930	6.318	29

**Table A24:** Summary of mixed-effects model results for Supraspinatus Tendon Thickness outcomes.

Mixed-effects model (REML)	Matching by factors:	Time and Side		
	Assume sphericity?	No		
	Alpha	0.05		
Fixed effects (type III)	*p* value	F (DFn, DFd)	Geisser-Greenhouse’s epsilon	
Time	<0.001	F (1.069, 61.99) = 14.24	0.5344	
Treatment	0.781	F (1, 116) = 0.078		
Side	<0.001	F (1, 58) = 28.06	1	
Time × Treatment	<0.001	F (1.069, 61.99) = 13.92	0.534	
Time × Side	<0.001	F (1.055, 61.17) = 16.13	0.527	
Treatment × Side	0.236	F (1, 116.0) = 1.418	1	
Time × Treatment × Side	<0.001	F (1.055, 61.17) = 12.53	0.527	
Random effects	SD	Variance		
Subject	0.442	0.196		
Subject × Time	0	0		
Subject × Side	0.478	0.229		
Residual	0.129	0.017		
Was the matching effective?	Chi-square, DF	648.5, 2	*p* value	<0.001
Compare each cell mean with every other cell mean				
Number of families		1		
Number of comparisons per family		66		

**Table A25:** Summary of the results from Bonferroni’s multiple comparison tests for Supraspinatus Tendon Thickness outcomes (only results related to the data shown in Table 6 are shown).

Bonferroni’s multiple comparisons test		Mean diff.	95% CI of diff.	Adjusted *p* Value			
BL	rESWT (a) vs. PTMs (a)	0.05	−0.67 to 0.77	>0.999			
	rESWT (c) vs. PTMs (c)	0.07	−0.51 to 0.65	>0.999			
	rESWT (a) vs. rESWT (c)	0.56	−0.02 to 1.15	0.070			
	PTMs (a) vs. PTMs (c)	0.58	0.04 to 1.12	0.023			
W6	rESWT (a) vs. PTMs (a)	−0.22	−0.82 to 0.38	0.975			
	rESWT (c) vs. PTMs (c)	0.06	−0.51 to 0.64	>0.999			
	rESWT (a) vs. rESWT (c)	0.30	−0.03 to 0.64	0.135			
	PTMs (a) vs. PTMs (c)	0.58	0.06 to 1.11	0.016			
W12	rESWT (a) vs. PTMs (a)	−0.27	−0.86 to 0.33	0.895			
	rESWT (c) vs. PTMs (c)	0.06	−0.52 to 0.64	>0.999			
	rESWT (a) vs. rESWT (c)	0.22	−0.09 to 0.54	0.815			
	PTMs (a) vs. PTMs (c)	0.55	0.03 to 1.08	0.027			
Test details		Mean 1	Mean 2	Mean diff.	SE of diff.	t	DF
BL	rESWT (a) vs. PTMs (a)	5.76	5.71	0.05	0.20	0.25	57.93
	rESWT (c) vs. PTMs (c)	5.20	5.13	0.07	0.16	0.43	57.77
	rESWT (a) vs. rESWT (c)	5.76	5.20	0.56	0.15	3.64	29.00
	PTMs (a) vs. PTMs (c)	5.71	5.13	0.58	0.14	4.06	29.00
W6	rESWT (a) vs. PTMs (a)	5.50	5.71	−0.22	0.17	1.30	51.30
	rESWT (c) vs. PTMs (c)	5.20	5.13	0.06	0.16	0.39	57.64
	rESWT (a) vs. rESWT (c)	5.50	5.20	0.30	0.09	3.39	29.00
	PTMs (a) vs. PTMs (c)	5.71	5.13	0.58	0.14	4.19	29.00
W12	rESWT (a) vs. PTMs (a)	5.42	5.69	−0.27	0.17	1.62	51.70
	rESWT (c) vs. PTMs (c)	5.20	5.14	0.06	0.16	0.38	57.73
	rESWT (a) vs. rESWT (c)	5.42	5.20	0.22	0.08	2.67	29.00
	PTMs (a) vs. PTMs (c)	5.69	5.14	0.55	0.14	3.99	29.00

**Table A26:** Summary of mixed-effects model results for Acromiohumeral Distance outcomes.

Mixed-effects model (REML)	Matching:	Stacked		
	Assume sphericity?	No		
	Alpha	0.05		
Fixed effects (type III)	*p* value	F (DFn, DFd)	Geisser-Greenhouse’s epsilon	
Time	<0.001	F (1.522, 88.28) = 11.80	0.761	
Treatment	0.608	F (1, 58) = 0.266		
Time × Treatment	<0.001	F (1.522, 88.28) = 13.56	0.761	
Random effects	SD	Variance		
Subject	0.846	0.715		
Residual	0.176	0.031		
Was the matching effective?	Chi-square, DF	307.1, 1	*p* value	<0.001
Difference between column means	Predicted mean of rESWT		10.93	
	Predicted mean of PTMs		10.82	
	Difference between predicted means		0.1133	
	SE of difference		0.2199	
	95% CI of difference		−0.327 to 0.553	
Data summary	Number of columns (Treatment)		2	
	Number of rows (Time)		3	
	Number of subjects (Subject)		60	
	Number of missing values		0	
Compare cell means with others in its row and its column				
Number of families		5		
Number of comparisons per row family		1		
Number of comparisons per column family		3		

**Table A27:** Summary of the results from Bonferroni’s multiple comparison tests for Acromiohumeral Distance outcomes.

Bonferroni’s multiple comparisons test		Mean diff.	95% CI of diff.	Adjusted *p* Value			
BL	rESWT vs. PTMs	−0.07167	−0.536 to 0.393	0.759			
W6	rESWT vs. PTMs	0.1577	−0.278 to 0.593	0.472			
W12	rESWT vs. PTMs	0.254	−0.184 to 0.692	0.251			
rESWT	BL vs. W6	−0.2473	−0.358 to −0.137	<0.001			
	BL vs. W12	−0.3003	−0.409 to −0.192	<0.001			
	W6 vs. W12	−0.053	−0.106 to −0.000	0.049			
PTMs	BL vs. W6	−0.018	−0.0428 to 0.007	0.227			
	BL vs. W12	0.02533	−0.138 to 0.188	>0.999			
	W6 vs. W12	0.04333	−0.118 to 0.205	>0.999			
Test details		Mean 1	Mean 2	Mean diff.	SE of diff.	t	DF
BL	rESWT vs. PTMs	10.75	10.82	−0.0717	0.232	0.300	58
W6	rESWT vs. PTMs	11	10.84	0.158	0.218	0.724	57.41
W12	rESWT vs. PTMs	11.05	10.8	0.254	0.219	1.16	57.42
rESWT	BL vs. W6	10.75	11	−0.247	0.043	5.70	29
	BL vs. W12	10.75	11.05	−0.300	0.043	7.013	29
	W6 vs. W12	11	11.05	−0.053	0.021	2.55	29
PTMs	BL vs. W6	10.82	10.84	−0.018	0.010	1.842	29
	BL vs. W12	10.82	10.8	0.025	0.064	0.395	29
	W6 vs. W12	10.84	10.8	0.043	0.064	0.683	29

## References

[1] Zhao J., Luo M., Liang G., Wu M., Pan J., Zeng L.F., Yang W., Liu J. (2021). Risk factors for supraspinatus tears: A meta-analysis of observational studies. Orthop. J. Sports Med..

[2] Vecchini E., Ricci M., Elena N., Gasperotti L., Cochetti A., Magnan B. (2022). Rotator cuff repair with single row technique provides satisfying clinical results despite consistent MRI retear rate. J. Orthop. Traumatol..

[3] Nyffeler R.W., Schenk N., Bissig P. (2021). Can a simple fall cause a rotator cuff tear? Literature review and biomechanical considerations. Int. Orthop..

[4] Lee J., Griepp D.W., Burgess C.J., Petrone B., Bitterman A.D., Cohn R.M. (2022). The AAOS 2019 clinical practice guidelines for the management of rotator cuff injuries are unbiased and incorporate a diverse body of literature. Arthrosc. Sports Med. Rehabil..

[5] Birinci Olgun T., Türkmen E., Altun S., Ziroglu N., Yeldan İ. (2024). Physiotherapist-supervised exercises versus physiotherapist-prescribed home exercises for treating partial thickness rotator cuff tears: A randomized controlled trial. J. Shoulder Elb. Surg..

[6] Nazligul T., Akpinar P., Aktas I., Unlu Ozkan F., Cagliyan Hartevioglu H. (2018). The effect of interferential current therapy on patients with subacromial impingement syndrome: A randomized, double-blind, sham-controlled study. Eur. J. Phys. Rehabil. Med..

[7] Yilmaz Kaysin M., Akpinar P., Aktas I., Unlü Ozkan F., Silte Karamanlioglu D., Cagliyan Hartevioglu H., Vural N. (2018). Effectiveness of shortwave diathermy for subacromial impingement syndrome and value of night pain for patient selection: A double-blinded, randomized, placebo-controlled trial. Am. J. Phys. Med. Rehabil..

[8] Afzalifard Z., Soltani A., Oskouei A.E. (2022). The Effects of magnet therapy on pain and disability in patients with shoulder impingement syndrome. Middle East J. Rehabil. Health Stud..

[9] Kurtaiş Gürsel Y., Ulus Y., Bilgiç A., Dinçer G., van der Heijden G.J. (2004). Adding ultrasound in the management of soft tissue disorders of the shoulder: A randomized placebo-controlled trial. Phys. Ther..

[10] Eslamian F., Shakouri S.K., Ghojazadeh M., Nobari O.E., Eftekharsadat B. (2012). Effects of low-level laser therapy in combination with physiotherapy in the management of rotator cuff tendinitis. Lasers Med. Sci..

[11] Galace de Freitas D., Marcondes F.B., Monteiro R.L., Rosa S.G., Maria de Moraes Barros Fucs P., Fukuda T.Y. (2014). Pulsed electromagnetic field and exercises in patients with shoulder impingement syndrome: A randomized, double-blind, placebo-controlled clinical trial. Arch. Phys. Med. Rehabil..

[12] Gunay Ucurum S., Kaya D.O., Kayali Y., Askin A., Tekindal M.A. (2018). Comparison of different electrotherapy methods and exercise therapy in shoulder impingement syndrome: A prospective randomized controlled trial. Acta Orthop. Traumatol. Turc..

[13] Calis H.T., Berberoglu N., Calis M. (2011). Are ultrasound, laser and exercise superior to each other in the treatment of subacromial impingement syndrome? A Randomized Clinical Trial. Eur. J. Phys. Rehabil. Med..

[14] Schmitz C., Császár N.B., Milz S., Schieker M., Maffulli N., Rompe J.D., Furia J.P. (2015). Efficacy and safety of extracorporeal shock wave therapy for orthopedic conditions: A systematic review on studies listed in the PEDro database. Br. Med. Bull..

[15] Reilly J.M., Bluman E., Tenforde A.S. (2018). Effect of shockwave treatment for management of upper and lower extremity musculoskeletal conditions: A narrative review. Phys. Med. Rehabil..

[16] Schroeder A.N., Tenforde A.S., Jelsing E.J. (2021). Extracorporeal shockwave therapy in the management of sports medicine injuries. Curr. Sports Med. Rep..

[17] Wuerfel T., Schmitz C., Jokinen L.L.J. (2022). The effects of the exposure of musculoskeletal tissue to extracorporeal shock waves. Biomedicines.

[18] Bjordal J.M., Demmink J.H., Ljunggren A.E. (2003). Tendon thickness and depth from skin for supraspinatus, common wrist and finger extensors, patellar and achilles tendons: Ultrasonography study. Physiotherapy.

[19] Császár N.B., Angstman N.B., Milz S., Sprecher C.M., Kobel P., Farhat M., Furia J.P., Schmitz C. (2015). Radial shock wave devices generate cavitation. PLoS ONE.

[20] Cacchio A., Paoloni M., Barile A., Don R., De Paulis F., Calvisi V., Ranavolo A., Frascarelli M., Santilli V., Spacca G. (2006). Effectiveness of radial shock-wave therapy for calcific tendinitis of the shoulder: Single-blind, randomized clinical study. Phys. Ther..

[21] Duymaz T., Sindel D. (2019). Comparison of radial extracorporeal shock wave therapy and traditional physiotherapy in rotator cuff calcific tendinitis treatment. Arch. Rheumatol..

[22] Li C., Li Z., Shi L., Wang P., Gao F., Sun W. (2021). Effectiveness of focused shockwave therapy versus radial shockwave therapy for noncalcific rotator cuff tendinopathies: A randomized clinical trial. BioMed Res. Int..

[23] Fuentes J.P., Armijo-Olivo S., Magee D.J., Gross D.P. (2010). Effectiveness of interferential current therapy in the management of musculoskeletal pain: A systematic review and meta-analysis. Phys. Ther..

[24] Atamaz F.C., Durmaz B., Baydar M., Demircioglu O.Y., Iyiyapici A., Kuran B., Oncel S., Sendur O.F. (2012). Comparison of the efficacy of transcutaneous electrical nerve stimulation, interferential currents, and shortwave diathermy in knee osteoarthritis: A double-blind, randomized, controlled, multicenter study. Arch. Phys. Med. Rehabil..

[25] Shah S., Farrow A., Esnouf A. (2007). Availability and use of electrotherapy devices: A survey. Int. J. Ther. Rehabil..

[26] Pope G.D., Mockett S.P., Wright J.P. (1995). A survey of electrotherapeutic modalities: Ownership and use in the NHS in England. Physiotherapy.

[27] Zwolińska J., Kasprzak M., Kielar A., Prokop M. (2024). Positive and negative effects of administering a magnetic field to patients with rheumatoid arthritis (RA). J. Clin. Med..

[28] World Medical Association (2025). World Medical Association Declaration of Helsinki: Ethical principles for medical research involving human participants. JAMA.

[29] Schulz K.F., Altman D.G., Moher D., CONSORT Group (2010). CONSORT 2010 Statement: Updated guidelines for reporting parallel group randomised trials. PLoS Med..

[30] Faul F., Erdfelder E., Buchner A., Lang A.G. (2009). Statistical power analyses using G*Power 3.1: Tests for correlation and regression analyses. Behav. Res. Methods.

[31] R Core Team (2021). R: A Language and Environment for Statistical Computing.

[32] Physiotherapy Evidence Database. https://pedro.org.au/.

[33] Haake M., Sattler A., Gross M.W., Schmitt J., Hildebrandt R., Müller H.H. (2001). Comparison of extracorporeal shockwave therapy (ESWT) with roentgen irradiation in supraspinatus tendon syndrome: A prospective randomized single-blind parallel-group study. Z. Orthop. Ihre Grenzgeb..

[34] Schmitt J., Haake M., Tosch A., Hildebrand R., Deike B., Griss P. (2001). Low-energy extracorporeal shock-wave treatment for tendinitis of the supraspinatus: A prospective, randomized study. J. Bone Joint Surg. Br..

[35] Speed C.A., Richards C., Nichols D., Burnet S., Wies J.T., Humphreys H., Hazleman B.L. (2002). Extracorporeal shock-wave therapy for tendinitis of the rotator cuff: A double-blind, randomized, controlled trial. J. Bone Joint Surg. Br..

[36] Schofer M.D., Hinrichs F., Peterlein C.D., Arendt M., Schmitt J. (2009). High- versus low-energy extracorporeal shock wave therapy for rotator cuff tendinopathy: A prospective, randomized, controlled study. Acta Orthop. Belg..

[37] Galasso O., Amelio E., Riccelli D.A., Gasparini G. (2012). Short-term outcomes of extracorporeal shock wave therapy for the treatment of chronic non-calcific supraspinatus tendinopathy: A double-blind, randomized, placebo-controlled trial. BMC Musculoskelet. Disord..

[38] Frizziero A., Vittadini F., Barazzuol M., Gasparre G., Finotti P., Meneghini A., Maffulli N., Masiero S. (2017). Extracorporeal shock wave therapy versus hyaluronic acid injection for the treatment of painful non-calcific rotator cuff tendinopathies: Preliminary results. J. Sports Med. Phys. Fit..

[39] Ebadi S., Karimzad Y., Aflakian N., Forogh B., Mansoori K., Babaei-Ghazani A. (2023). Extracorporeal shock wave therapy versus corticosteroid injection for the treatment of non-calcific rotator cuff tendinopathies: A randomized trial. Curr. Orthop. Pract..

[40] American Academy of Orthopaedic Surgeons (AAOS) (2022). Rotator Cuff and Shoulder Conditioning Program.

[41] Angst F., Schwyzer H.K., Aeschlimann A., Simmen B.R., Goldhahn J. (2011). Measures of adult shoulder function: Disabilities of the Arm, Shoulder, and Hand Questionnaire (DASH) and its short version (QuickDASH), Shoulder Pain and Disability Index (SPADI), American Shoulder and Elbow Surgeons (ASES) Society Standardized Shoulder Assessment Form, Constant (Murley) Score (CS), Simple Shoulder Test (SST), Oxford Shoulder Score (OSS), Shoulder Disability Questionnaire (SDQ), and Western Ontario Shoulder Instability Index (WOSI). Arthritis Care Res..

[42] Wylie J.D., Beckmann J.T., Granger E., Tashjian R.Z. (2014). Functional outcomes assessment in shoulder surgery. World J. Orthop..

[43] Tashjian R.Z., Deloach J., Green A., Porucznik C.A., Powell A.P. (2010). Minimal clinically important differences in ASES and simple shoulder test scores after nonoperative treatment of rotator cuff disease. J. Bone Joint Surg. Am..

[44] Jain N.B., Wilcox R.B., Katz J.N., Higgins L.D. (2013). Clinical examination of the rotator cuff. Phys. Med. Rehabil..

[45] Dean A.G., Sullivan K.M., Soe M.M. Open Source Epidemiologic Statistics for Public Health (OpenEpi). https://www.openepi.com/Power/PowerMean.htm.

[46] Austin P.C. (2009). Balance diagnostics for comparing the distribution of baseline covariates between treatment groups in propensity-score matched samples. Stat. Med..

[47] Waugh C.M., Morrissey D., Jones E., Riley G.P., Langberg H., Screen H.R. (2015). In vivo biological response to extracorporeal shockwave therapy in human tendinopathy. Eur. Cells Mater..

[48] Hochstrasser T., Frank H.G., Schmitz C. (2016). Dose-dependent and cell type-specific cell death and proliferation following in vitro exposure to radial extracorporeal shock waves. Sci. Rep..

[49] Chen Y.J., Wang C.J., Yang K.D., Kuo Y.R., Huang H.C., Huang Y.T., Sun Y.C., Wang F.S. (2004). Extracorporeal shock waves promote healing of collagenase-induced Achilles tendinitis and increase TGF-β1 and IGF-I expression. J. Orthop. Res..

[50] Yoo S.D., Choi S., Lee G.J., Chon J., Jeong Y.S., Park H.K., Kim H.S. (2012). Effects of extracorporeal shockwave therapy on nanostructural and biomechanical responses in the collagenase-induced Achilles tendinitis animal model. Lasers Med. Sci..

[51] Vetrano M., d’Alessandro F., Torrisi M.R., Ferretti A., Vulpiani M.C., Visco V. (2011). Extracorporeal shock wave therapy promotes cell proliferation and collagen synthesis of primary cultured human tenocytes. Knee Surg. Sports Traumatol. Arthrosc..

[52] Cheng Y., Zhang J., Cai Y. (2016). Utility of ultrasonography in assessing the effectiveness of extracorporeal shock wave therapy in insertional Achilles tendinopathy. BioMed Res. Int..

[53] El-Mallah N.A., Elattar A.N. (2020). Effect of extracorporeal shock wave therapy versus mesotherapy in the treatment of achilles tendinopathy: A randomized clinical trial and ultrasonographic assessment. Egypt. Rheumatol. Rehabil..

[54] Notarnicola A., Moretti B. (2012). The biological effects of extracorporeal shock wave therapy (ESWT) on tendon tissue. Muscles Ligaments Tendons J..

[55] Visco V., Vulpiani M.C., Torrisi M.R., Ferretti A., Pavan A., Vetrano M. (2014). Experimental studies on the biological effects of extracorporeal shock wave therapy on tendon models: A review of the literature. Muscles Ligaments Tendons J..

[56] Poenaru D., Sandulescu M.I., Cinteza D. (2022). Biological effects of extracorporeal shockwave therapy in tendons: A systematic review. Biomed. Rep..

[57] Kim H., Kim B., Shim J., Kwon H., Jung J. (2014). Comparative analysis of acromiohumeral distances according to the locations of the arms and humeral rotation. J. Phys. Ther. Sci..

[58] Park H.J., Lee S.Y., Choi Y.J., Park J.H., Kim E. (2018). Association between subacromial impingement and acromiohumeral distance on MRI. Iran. J. Radiol..

[59] Xu M., Li Z., Zhou Y., Ji B., Tian S., Chen G. (2020). Correlation between acromiohumeral distance and the severity of supraspinatus tendon tear by ultrasound imaging in a Chinese population. BMC Musculoskelet. Disord..

[60] Maier M., Averbeck B., Milz S., Refior H.J., Schmitz C. (2003). Substance P and prostaglandin E_2_ release after shock wave application to the rabbit femur. Clin. Orthop. Relat. Res..

[61] ElGendy M.H., Mazen M.M., Saied A.M., ElMeligie M.M., Aneis Y. (2023). Extracorporeal shock wave therapy vs. corticosteroid local injection in shoulder impingement syndrome: A three-arm randomized controlled trial. Am. J. Phys. Med. Rehabil..

[62] Rompe J.D., Furia J., Maffulli N. (2009). Eccentric loading versus eccentric loading plus shock-wave treatment for midportion Achilles tendinopathy: A randomized controlled trial. Am. J. Sports Med..

